# Evolution of the Noncoding Features of Sea Snake Mitochondrial Genomes within Elapidae

**DOI:** 10.3390/genes13081470

**Published:** 2022-08-17

**Authors:** Xiakena Xiaokaiti, Yasuyuki Hashiguchi, Hidetoshi Ota, Yoshinori Kumazawa

**Affiliations:** 1 Department of Information and Basic Science and Research Center for Biological Diversity, Graduate School of Science, Nagoya City University, Nagoya 467-8501, Japan; 2 Department of Biology, Faculty of Medicine, Osaka Medical and Pharmaceutical University, Takatsuki 569-0801, Japan; 3 Institute of Natural and Environmental Sciences, University of Hyogo, and Museum of Nature and Human Activities, Sanda 669-1546, Japan

**Keywords:** control region, tandem repeat, Nanopore sequencing, light-strand replication origin

## Abstract

Mitochondrial genomes of four elapid snakes (three marine species [*Emydocephalus ijimae*, *Hydrophis ornatus*, and *Hydrophis melanocephalus*], and one terrestrial species [*Sinomicrurus japonicus*]) were completely sequenced by a combination of Sanger sequencing, next-generation sequencing and Nanopore sequencing. Nanopore sequencing was especially effective in accurately reading through long tandem repeats in these genomes. This led us to show that major noncoding regions in the mitochondrial genomes of those three sea snakes contain considerably long tandem duplications, unlike the mitochondrial genomes previously reported for same and other sea snake species. We also found a transposition of the light-strand replication origin within a tRNA gene cluster for the three sea snakes. This change can be explained by the Tandem Duplication—Random Loss model, which was further supported by remnant intervening sequences between tRNA genes. Mitochondrial genomes of true snakes (Alethinophidia) have been shown to contain duplicate major noncoding regions, each of which includes the control region necessary for regulating the heavy-strand replication and transcription from both strands. However, the control region completely disappeared from one of the two major noncoding regions for two *Hydrophis* sea snakes, posing evolutionary questions on the roles of duplicate control regions in snake mitochondrial genomes. The timing and molecular mechanisms for these changes are discussed based on the elapid phylogeny.

## 1. Introduction

Vertebrate mitochondrial DNAs (mtDNAs) are maternally-inherited circular double-stranded DNAs of approximately 17 kbp in length and they compactly encode 37 genes for 2 rRNAs, 22 tRNAs, and 13 respiratory-chain proteins, as well as an approximately 1-kbp major non-coding region (MNCR) [1,2,3]. The MNCR includes the control region containing signals that initiate the transcription of the heavy and light strands (H and L strands, respectively) and the replication of the H strand of the mitochondrial genomes [1,4]. The control region typically contains conserved sequence blocks (CSBs) and the core termination-associated sequence (coreTAS) which are believed to serve as common motifs in regulating the transcription and/or replication in various vertebrates [5,6,7,8,9,10].

**Figure 1:**
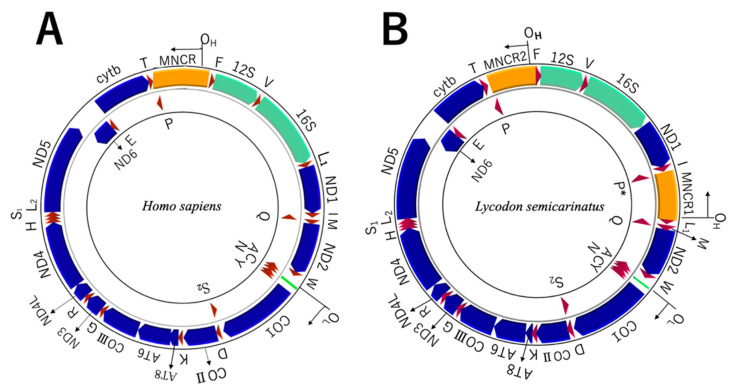
Gene arrangements of mitochondrial genomes of (**A**) the human [1] and (**B**) the colubrid snake *Lycodon semicarinatus* [12]. Genes encoded by the heavy strand are shown in the outer circle while those encoded by the light strand are shown in the inner circle. Abbreviations for genes are: 12S and 16S, 12S and 16S rRNA genes; CO I–III, cytochrome oxidase subunits I–III genes; ND1–6 and 4L, NADH dehydrogenase subunits 1–6 and 4L genes; AT8 and AT6, ATPase subunits 8 and 6 genes; and cytb, cytochrome *b* gene. Transfer RNA genes are denoted by one letter of the corresponding amino acids. L_1_, L_2_, S_1_, S_2_ and p* represent genes for tRNA^Leu^ (UUR), tRNA^Leu^ (CUN), tRNA^Ser^ (AGY), tRNA^Ser^ (UCN), and a pseudogene for tRNA^Pro^, respectively. Abbreviations for noncoding features are: MNCR, major noncoding region; O_H_, heavy-strand replication origin; and O_L_, light-strand replication origin.

The gene arrangement of human mtDNA (Figure 1A), for which the complete mtDNA sequence was first reported for animals [1], was subsequently found to be conserved among many species of mammals, reptiles, amphibians, and fish, thereby being occasionally called the typical vertebrate mtDNA gene arrangement (reviewed in [3,11]). However, there have also been many known examples of partial gene rearrangements in various vertebrate groups, and both coding and noncoding features of vertebrate mitochondrial genomes are not fixed but evolving [3,11].

For snakes, the entire mtDNA sequence was first determined for the colubrid *Lycodon semicarinatus* (as *Dinodon semicarinatus*) [12], and it was found to contain two identical control region sequences in duplicated MNCRs (Figure 1B). Since then, complete mtDNA sequences for more than 100 snake species have been deposited to the public database to show that mitochondrial genomes for true snakes (Alethinophidia), except for the primitive blind snakes (Scolecophidia), commonly contain duplicate MNCRs, one between the tRNA^Ile^ and tRNA^Leu^ (UUR) genes (hereafter named MNCR1) and the other between the tRNA^Pro^ and tRNA^Phe^ genes (MNCR2) (see, e.g., [13,14,15]). The control region sequences in the MNCR1 and MNCR2 were identical or nearly identical when compared within a species, but they were divergent between species. Therefore, it was inferred that the duplicate control region sequences have evolved concertedly by mechanisms, such as frequent gene conversion [12,13,14,15]. Duplication of the control region sequences within a mtDNA molecule have actually been found from diverse vertebrate taxa of fish, amphibians, birds, and reptiles [16,17,18,19,20,21,22]. However, the long (>70 million years) [13] persistence of the duplicate state of the control regions and their concerted evolution in the snake mtDNAs seems conspicuous in posing an evolutionary question why apparently redundant duplicate sequences have been maintained for such a long period of time in snake mitochondrial genomes.

In the process of sequencing and analyzing the mitochondrial genomes of various vertebrates, we encountered sea snake mtDNAs that appeared to have, unlike previous reports on some sea snake mtDNAs, exceptionally long MNCR regions. With the aid of the Nanopore long-read technology (reviewed in [23]), we read through these long mtDNAs and found some changes in noncoding regions including the disappearance of the control region from one of the MNCRs. In this work, we describe characteristics found for complete mitochondrial genomes of four elapid snakes and discuss evolutionary implications of these findings.

## 2. Materials and Methods

### 2.1. Samples

Tissue samples of four Japanese elapid snakes were obtained from museums or a specimen depository in Japan (Table 1). Three of them (*Emydocephalus ijimae*, *Hydrophis ornatus*, and *Hydrophis melanocephalus*) were sea snakes with completely aquatic life-histories from the subfamily Hydrophiinae and one remaining species (*Sinomicrurus japonicus*) was a terrestrial Asian coral snake from Elapinae. Elapidae is a family of poisonous snakes with 53 genera and 390 species [24] and typically divided into two subfamilies (Elapinae and Hydrophiinae) [25]. Elapinae consists of terrestrial species further divided into tribes Hemibungarini (cobras) and Calliophini (coral snakes) [26]. Hydrophiinae consists of Hydrophiini (sea snakes), Laticaudini (sea kraits), and Australasian terrestrial species [27]. *Hydrophis* is the most species-rich (49 species) genus of Hydrophiini. DNA extraction was performed using the DNeasy Blood and Tissue Kit (Qiagen) from muscle tissue.

### 2.2. Sequencing of mtDNAs

First, MNCR1 and MNCR2 regions were amplified with flanking reptile-oriented primers (Table S1). Tks Gflex DNA polymerase (Takara Bio, Kusatsu, Japan) was used as the enzyme. The thermal cycling condition was 94 °C for 1 min followed by 30 cycles of 98 °C for 10 s, 55 °C for 15 s, and 68 °C for 2 min. The BigDye Terminator v3.1 Cycle Sequencing Kit (Thermo Fisher Scientific, Waltham, MA, USA) was used for the dye terminator sequencing of the amplified products for both directions by the Sanger method with an Applied Biosystems Genetic Analyzer 3500 (Thermo Fisher Scientific, Waltham, MA, USA). The primer walking strategy was taken to read the amplified regions from both ends until unique primers could no longer be designed against repeated sequences within the MNCRs.

Second, species-specific primers (Table S2) were designed in the sequences determined above, and the long PCR was performed to cover the entire mtDNAs with a few amplified fragments. PrimeStar GXL DNA polymerase (Takara Bio, Kusatsu, Japan) was used as the enzyme and 30–35 cycles of 98 °C for 10 s and 68 °C for 12 min were conducted for the long PCR. The products were mixed in equimolar ratios within each species and multiplexed DNA libraries were created using the NEBNext Ultra II FS DNA Library Prep Kit for Illumina (NEB). A 101-bp paired-end sequencing run was then performed using an Illumina MiSeq next-generation sequencer with the MiSeq Reagent Nano Kit v2 (Illumina). Subsequently, Solexa QA software [28] was used to remove low quality bases in each read and trim short reads, followed by the assembly with Trinity [29] to give rise to consensus contig sequences that cover the entire amplified regions by the long PCR.

Third, the Nanopore long-read sequencing (reviewed in [23]) was applied to determine the whole MNCR region sequences that include long repeats. The primers described above (Table S1) were used to amplify MNCR regions for three sea snakes and an intervening sequence between tRNA^Cys^ and tRNA^Tyr^ genes for the coral snake. Tks Gflex DNA polymerase (Takara Bio, Kusatsu, Japan) was used as the enzyme with the above-mentioned thermal cycling condition. 0.5% agarose gel electrophoresis was used to measure the length of amplified products. Each amplified product was cut from the agarose gel, purified, and quantified with the Qubit^TM^ dsDNA HS Assay Kit and the Qubit fluorometer (Thermo Fisher Scientific, Waltham, MA, USA). Multiplexed DNA libraries were created using the Ligation Sequencing Kit and Native Barcoding Expansion 1–12 (Oxford Nanopore Technologies, Oxford, UK). Nanopore sequencing was carried out with the Minion MK 1B device (Oxford Nanopore Technologies, Oxford, UK) according to the manufacturer’s instructions. Base calls were performed using MinKNOW 4.1.22 (Oxford Nanopore Technologies, Oxford, UK) in the high precision base call mode. Among reads assigned to each sample after demultiplexing, only reads that were within ±10% in the nucleotide length compared to the Long PCR product size were selected as those that may correspond to the full length of the PCR product. These selected reads were then assembled with canu 2.2 software [30] to correct potential errors associated with the Nanopore sequencing with sufficient read depths at each nucleotide position.

Finally, the sequences determined by the Sanger method, Illumina next-generation sequencing method, and Nanopore sequencing method were assembled using Sequencher v4.8 (Gene Codes, Ann Arbor, MI, USA) to complete the entire mtDNA sequence for each species. These new sequences were deposited to DDBJ database, as a part of the International Nucleotide Sequence Database (INSD), under accession numbers shown in Table 1.

### 2.3. Annotation

Each protein gene was identified by searching for open reading frames and comparing translated amino acid sequences with counterparts from other snake species. Some of the termination codons were not encoded in the mtDNA as in other vertebrates and appeared to arise by polyadenylation when the transcript underwent the processing [31].

Transfer RNA genes were identified by referring to the secondary structures of mitochondrial tRNAs [32] and sequence similarity to counterparts of other snake species. Ribosomal RNA genes were identified by their sequence similarities to the counterparts of other snake species. The stable stem-and-loop structures for the light-strand replication origin (O_L_) were identified with DNASIS-MAC v3.5 (Hitachi Software Engineering, Tokyo, Japan). Conserved regions in the control region (e.g., CSB and coreTAS) were identified by searching for common motif sequences for them. Repeat sequences were identified using the Multiple Sequence Homology Plot option of DNASIS-MAC v3.5.

### 2.4. Phylogenetic Analysis

Phylogenetic analysis was performed by the maximum likelihood (ML) method using nucleotide sequences of all 37 genes (third codon positions deleted for protein genes) or amino acid sequences of all 13 protein genes. ML analysis was performed using the RAxML v8.2.8 [33]. For the model selection and other analytic procedures, we referred to ModelGenerator v.0.85 [34] and the manual of RAxML v8.2.8. The GTRGAMMA model was employed in the nucleotide sequence analysis, and the ML tree was selected after five independent runs with random seeds. The bootstrap probability at each node was assessed by 10,000 bootstrap replications using the GTRCAT model. For the analysis using amino acid sequences, the PROTGAMMAMTREVF model was selected, and the ML tree was selected after 5 independent runs. The bootstrap probability at each node was also assessed by 5000 bootstrap replications using the PROTCATMTREVF model.

## 3. Results

### 3.1. Sequencing Snake Mitochondrial Genomes

In this study, we used a combination of Sanger sequencing, next-generation sequencing, and Nanopore sequencing to complete the sequencing of entire mtDNA molecules for *E. ijimae*, *H. ornatus*, *H. melanocephalus*, and *S. japonicus* (Table 1). As described by Dong and Kumazawa [14], we first used reptile-oriented primers (Table S1) to amplify and sequence MNCR regions between the NADH dehydrogenase subunit 1 and tRNA^Met^ genes (MNCR1), and between the tRNA^Thr^ and 12S rRNA genes (MNCR2). Sanger-sequencing of the amplified products from both ends followed by the primer walking provided complete sequences for both MNCR1 and MNCR2 regions for *S. japonicus*, which were relatively short in length (approximately 1kbp; Table 1) and did not include repetitive sequences. However, for the other three species, long tandem repeats within the MNCR regions hampered their complete sequencing by the Sanger method.

Species-specific primers for the long PCR (Table S2) were then designed based on the unique nucleotide sequences at both ends of the MNCR1 and MNCR2 regions. Mitochondrial DNA regions between MNCR1 and MNCR2 were successfully amplified with these primers for *E. ijimae*, *H. ornatus*, and *H. melanocephalus*, but not for *S. japonicus*. We therefore designed additional species-specific primers against cytochrome oxidase subunit III gene and amplified *S. japonicus* mtDNA with three long-PCR fragments (Table S2). Next-generation sequencing of short reads originating from these long-PCR products by an Illumina MiSeq sequencer yielded 190,000–480,000 reads per species and the assembly of these reads gave rise to single contigs covering targeted regions for each species, except for the 9.5-kbp fragment of *S. japonicus* (Table S2). We knew that this fragment included exceptionally long tandem repeats between the tRNA^Cys^ and tRNA^Tyr^ genes, which must be sequenced in another way.

Nanopore sequencing was used to complete the sequencing of long tandem repeat regions. The two MNCR regions for *E. ijimae*, *H. ornatus*, and *H. melanocephalus*, as well as the region between the tRNA^Cys^ and tRNA^Tyr^ genes for *S. japonicus* were amplified with primers listed in Table S1. The Nanopore sequencing of these amplified products produced 100,000–480,000 reads, and the assembly of many error-prone Nanopore reads provided single continuous sequences for each amplified product with sizes estimated from 0.5% agarose gel electrophoresis (Table S1). These sequences were assembled with those for other mtDNA regions already determined by the Sanger and next-generation methods to provide the complete full-length mtDNA sequences of the four snake species (Table 1). However, it should be noted that the long-PCR amplification of the MNCR2 region for *H. melanocephalus* produced two bands in the agarose gel electrophoresis (data not shown). Nanopore sequencing of each product recovered from the gel revealed that these products differed in the number of tandem repeats (i.e., five vs. four repeats of the 0.6-kbp repetitive unit in 3.4 kbp and 2.8 kbp products, respectively; Table S1). This could be recognized as the heteroplasmic state of mtDNA in the repetitive number of tandem repeats.

### 3.2. Encoded Genes

**Figure 2:**
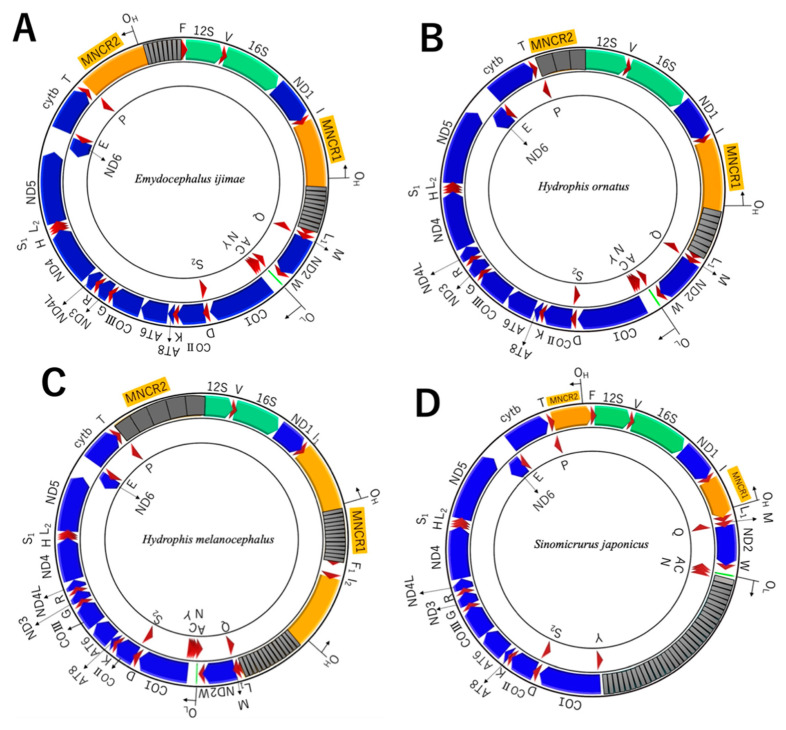
Gene arrangements of mitochondrial genomes of the four snake species sequenced in this study. (**A**): *Emydocephalus ijimae*, (**B**): *Hydrophis ornatus*, (**C**): *Hydrophis melanocephalus* and (**D**): *Sinomicrurus japonicus*. For abbreviations of genes and noncoding features, see the legend of Figure 1. Tandem gray boxes stand for tandem repeats with the indicated number of repeating times.

The complete mtDNA sequences thus determined for the four species ranged in size from 19.7 kbp to 26.3 kbp (Table 1) and were relatively long compared to those of other snakes (e.g., 17,191 bp for *Lycodon semicarinatus* as *Dinodon semicarinatus*; [12]). By gene annotation 13 protein genes and 2 rRNA genes were identified for each species like most other vertebrates (Figure 2; Tables S3–S6). With regard to tRNA genes, the usual 22 tRNA genes were identified for *E. ijimae* and *S. japonicus*, while the other two species showed duplicate copies for some tRNA genes in addition to the usual 22 tRNA genes. Three copies of the tRNA^Phe^ gene were found in *H. ornatus* mtDNA, while two copies of the tRNA^Ile^ gene, five copies of the tRNA^Pro^ gene, and six copies of the tRNA^Phe^ gene were present in *H. melanocephalus* mtDNA. Some of these duplicated tRNA genes could be pseudogenic (see Section 3.4 for details). These protein, rRNA, and tRNA genes were compactly arranged in the mitochondrial genomes with minimum gap nucleotides (Tables S3–S6).

### 3.3. Non-Coding Regions in WANCY

The mitochondrial genomes of four elapid snakes showed some changes in non-coding regions. Within the WANCY tRNA gene cluster, a characteristic stem-and-loop structure for O_L_ [35] usually exists between the tRNA^Asn^ and tRNA^Cys^ genes for most vertebrates (Figure 1). The mitochondrial genome of *S. japonicus* also had the O_L_ at this position, but those of *E. ijimae*, *H. ornatus*, and *H. melanocephalus* had the O_L_ between the tRNA^Ala^ and tRNA^Asn^ genes (Tables S3–S6). Spacers between the tRNA^Ala^ and tRNA^Asn^ genes were 124 bp, 146 bp, and 138 bp, respectively, for these latter species. The stem-and-loop structure for the O_L_ occupied 34–40 bp in the middle of these spacers (Figure S1). The remaining parts of the spacers, especially ~25 bp immediately 5′ to the O_L_ and ~10 bp immediately 3′ to the O_L_, retained certain sequence similarities between the species but did not show any recognizable sequence similarity to other coding and noncoding regions of mtDNAs.

In the WANCY region of *S. japonicus*, there was an exceptionally long (4422 bp) intervening sequence between the tRNA^Cys^ and tRNA^Tyr^ genes (Figure 2). This insertion included 32 tandem repeats of a 112 bp unit that did not show any recognizable sequence similarity to other coding and noncoding regions of *S. japonicus* mtDNA (Figure S1).

### 3.4. Non-Coding Regions in MNCRs

MNCR1 and MNCR2 of *S. japonicus* mtDNA were 1028 and 1008 bp long, respectively (Table 1) and did not contain repeated sequences in them. These are standard sizes for MNCR regions of many snakes: e.g., 0.9–1.2 kbp for *Lycodon semicarinatus* (Colubridae), *Ovophis okinavensis* (Viperidae), *Python regius* (Pythonidae), and *Acrochordus granulatus* (Acrochordidae) [12,13,14]. The C-rich, coreTAS, CSB-I and CSB-III sequences were identified in both MNCR1 and MNCR2, indicating the presence of the control regions (Table S6; Figures S2 and S3). Alignable MNCR1 and MNCR2 sequences (998 bp) were found to differ by 9 nucleotides. This level of difference has been observed between the duplicated control regions of some snake mitochondrial genomes (e.g., *Boa constrictor* [Boidae]) [14].

**Figure 3:**
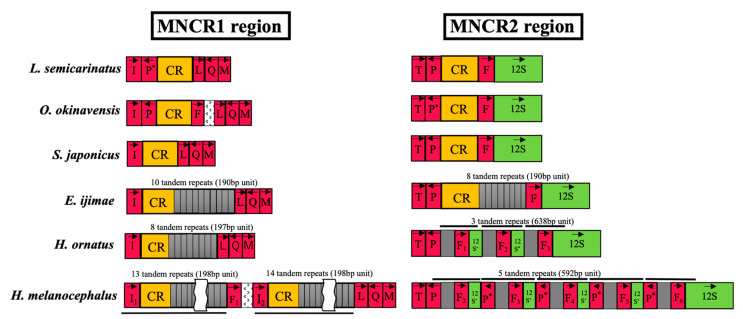
Comparison of features in MNCR1 and MNCR2 regions between several snakes: the colubrid *Lycodon semicarinatus* [12], the viperid *Ovophis okinavensis* [14], and four elapid snakes sequenced in this study. Abbreviations of genes and noncoding features are similar to those in Figure 1 and Figure 2, except that directions of encoded genes are shown by an arrow. CR stands for the control region and 12S* represents the 5’-end partial sequence for the 12S rRNA gene. When there are multiple copies for the tRNA^Phe^ and tRNA^Ile^ genes including potential pseudogenes, they are discriminated by the numbering.

In contrast, the MNCRs of *E. ijimae*, *H. ornatus*, and *H. melanocephalus* were rather long (1.7–8.3 kbp; Table 1). For *E. ijimae*, the C-rich, coreTAS, CSB-I, and CSB-III sequences were identified in both MNCR1 and MNCR2 (Table S3; Figures S2 and S3), indicating the existence of the control regions. Alignable MNCR1 and MNCR2 sequences excluding repeated regions (962 bp) were found to be identical. The complete sequence matching between two duplicate control regions has been observed in mitochondrial genomes of many snakes (e.g., *Ovophis okinavensis* and *Acrochordus granulatus*) [14]. However, both MNCR1 and MNCR2 of *E. ijimae* contained long tandem repetition of a 190 bp unit at the 3’ end of the control regions, which was responsible for the expansion of the *E. ijimae* MNCRs (Figure 3). This 190-bp repeat unit was identical between MNCR1 and MNCR2 and repeated 10 times in MNCR1 and 8 times in MNCR2.

MNCR1 regions for both *H. ornatus* and *H. melanocephalus* contained the control region as the C-rich, coreTAS, CSB-I, and CSB-III sequences were identified (Tables S4 and S5; Figures S2 and S3). The control region in *H. ornatus* MNCR1 was followed by 8 tandem repeats of a 197-bp repeat unit (Figure 3). The MNCR1 of *H. melanocephalus* was very long (8330 bp; Table 1), in which a long DNA segment containing the tRNA^Ile^ gene, the control region, and tandem repeats of a 198-bp unit, was duplicated with the tRNA^Phe^ gene between them (Figure 3). Sequence comparison of the duplicated tRNA^Ile^ genes (I_1_ and I_2_ in Figure 3) revealed a two-nucleotide difference between them, with a slightly less stable acceptor stem in the secondary structure of the I_2_ copy (Figure S4). Thus, functional tRNA^Ile^ molecules may be produced primarily from the I_1_ copy, though they may also be produced from the I_2_ copy.

MNCR2 regions of *H. ornatus* and *H. melanocephalus* did not contain any conserved blocks such as the C-rich, coreTAS and CSBs, showing no sequence similarity to the control regions of other snake species (Figure S3). At *H. ornatus* MNCR2, there were three tandem repetitions of a 638-bp unit that contained the tRNA^Phe^ gene and a 5′-end partial sequence of the 12S rRNA gene (Figure 3). Thus, three tRNA^Phe^ gene copies occur in *H. ornatus* MNCR2. Comparison of their sequences and secondary structures (Figure S5) showed that the third copy (F_3_ in Figure 3) may have slightly higher D-stem stability in the secondary structure than the other two copies (F_1_ and F_2_ in Figure 3). It is therefore likely that functional tRNA^Phe^ molecules are generated primarily from the F_3_ copy, but we cannot rule out the possibility that they are also made from the other copies.

At *H. melanocephalus* MNCR2, there were five tandem repetitions of a 592-bp unit that contained a half part of the tRNA^Pro^ gene (i.e., pseudo tRNA^Pro^ gene), the tRNA^Phe^ gene, and a 5′-end partial sequence of the 12S rRNA gene (Figure 3). Thus, a legitimate copy of the tRNA^Pro^ gene, as well as four copies of the pseudo tRNA^Pro^ gene occur at MNCR2 of this species. With respect to the tRNA^Phe^ genes, five copies are found at MNCR2 in addition to one more copy at MNCR1. Comparison of their sequences and secondary structures (Figure S5) showed that all copies at MNCR2 could be pseudogenic as they had eight nucleotides for the anticodon loop. Only the copy at MNCR1 (denoted F_1_ in Figure 3) could assume a secondary structure with standard seven nucleotides for the anticodon loop, suggesting that this is the functional tRNA^Phe^ gene in *H. melanocephalus* mtDNA.

## 4. Discussion

### 4.1. Accuracy in Sequencing Mitochondrial Genomes

Methods for sequencing mitochondrial genomes have been changed by the improvement of sequencing methods to attain the accuracy and throughput. Sanger sequencing is a steady and very accurate method but the maximum length of accurately readable bases by a standard capillary DNA sequencer is up to 700–900 bp and its experimental throughput may not be satisfactory for sequencing multiple mitochondrial genomes in parallel. Next-generation sequencing represented by the Illumina sequencing protocol provides high throughput and read depths enough to cover ~20-kbp mtDNAs but this approach has a critical disadvantage in assembling short (100–300 bp) reads obtained from highly repetitive regions. Nanopore long-read sequencing can read through the long repetitive sequences in principle. However, it is a relatively new approach for use of de novo sequencing mtDNAs (e.g., [36,37,38,39]) and its utility in sequencing long repeat regions accurately has not been well-established. The present study demonstrated the usefulness of the Nanopore method in completely sequencing sea snake mtDNAs with >8 kbp of repetitive regions. This method will contribute to the complete sequencing of other repeat-prone mtDNAs in future.

During the course of the present study, complete mtDNA sequences were reported for three semiaquatic sea snakes of the genus *Laticauda* and several fully aquatic sea snakes of the genera *Emydocephalus* and *Hydrophis* (all in the subfamily Hydrophiinae) [40,41,42,43,44,45]. Among them, *E. ijimae* (Accession No. MK775531; 18,259 bp) [42] and *H. melanocephalus* (Accession No. MK775532; 17,182 bp) [43] overlapped with the species sequenced in this study. However, mtDNA sequences reported in these studies for *E. ijimae* and *H. melanocephalus* were much shorter than those determined in this study (Table 1). Sequence comparison revealed that their sequences were similar to ours at non-repetitive regions but contained no or only short tandem duplication within MNCRs (Table S7). Long tandem repeats within MNCRs were also absent from most mtDNA sequences reported by other investigators for the genera *Hydrophis* and *Laticauda* (Table S7).

Unfortunately, the above-cited papers from other researchers were generally short and did not contain sufficient description of how accurate sequencing of repetitive regions in MNCRs was warranted. Taken together, we doubt the accuracy of MNCR sequences determined by the other researchers. In the following sections, we therefore develop discussion by incorporating only non-MNCR sequences reported by these authors. To the best of our knowledge, these papers did not describe characteristics of sea snake mtDNAs, such as the rearrangement of O_L_ within WANCY and disappearance of the control region from MNCR2. We will focus on molecular evolution of these features based on the snake phylogeny.

### 4.2. Rearrangement of L-Strand Replication Origin

**Figure 4:**
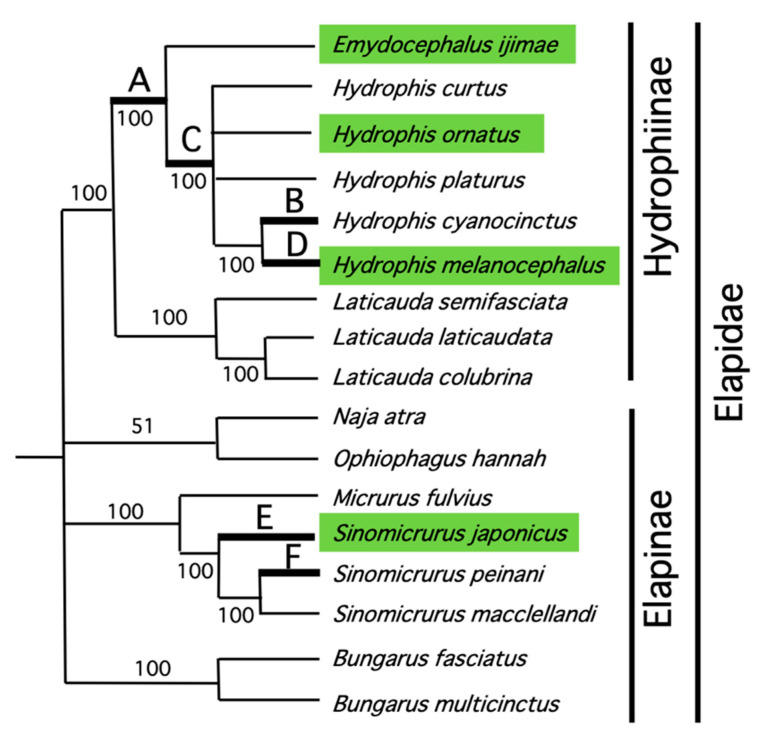
Timing of changes in mtDNA structural features in Elapidae. On a strict consensus tree between the ML trees constructed using nucleotide and amino acid sequences (see Figures S6 and S7, respectively), the (**A**–**F**) symbols indicate branches where changes in mtDNA features probably occurred as inferred by the maximum-parsimony criterion in change numbers. These changes are: (**A**), translocation of O_L_ from between tRNA^Asn^ and tRNA^Cys^ genes to between tRNA^Ala^ and tRNA^Asn^ genes; (**B**), reversal of the O_L_ translocation from between tRNA^Ala^ and tRNA^Asn^ genes to between tRNA^Asn^ and tRNA^Cys^ genes; (**C**), loss of the control region from MNCR2; (**D**), duplication of the control region within MNCR1; and (**E**,**F**), independent insertions of long tandem repeats between tRNA^Cys^ and tRNA^Tyr^ genes. Numbers on branches represent bootstrap probabilities on the ML tree constructed using nucleotide sequences (Figure S6). Species highlighted in green represent the four species sequenced in this study.

The mtDNA sequences of the four snakes obtained in this study, as well as those of 17 other snake species downloaded from the INSD database (Table S8), were used to construct ML trees. The ML trees constructed using nucleotide sequences of all 37 genes (Figure S6) and amino acid sequences of 13 protein genes (Figure S7) were very similar, except for the phylogenetic placement of some genera in the tribe Hemibungarini and inter-species relationships within the genus *Hydrophis*. Based on a strict consensus tree between these ML trees, the timing of the emergence/disappearance of features in the mitochondrial genomes was inferred using the maximum parsimony criterion for relevant events (Figure 4).

In addition to *E. ijimae*, *H. ornatus*, and *H. melanocephalus* sequenced in this study, two other species (*H. curtus* and *H. platurus*) had the O_L_ between the tRNA^Ala^ and tRNA^Asn^ genes. Although a congeneric species (*H. cyanocinctus*) retained the typical position for the O_L_ (i.e., between tRNA^Asn^ and tRNA^Cys^ genes), the spacer between the tRNA^Ala^ and tRNA^Asn^ genes showed sequence similarity between *H. cyanocinctus* and other *Emydocephalus*/*Hydrophis* species (Figure S1). Under this circumstance, the transposition of O_L_ from between tRNA^Asn^ and tRNA^Cys^ genes to between tRNA^Ala^ and tRNA^Asn^ genes presumably occurred once in the common ancestor of *Emydocephalus* and *Hydrophis* on a branch A of Figure 4, and then a reversal to the original position likely occurred on a lineage leading to *H. cyanocinctus* (branch B). It should be noted that the phylogenetic clustering of *H. melanocephalus* and *H. cyanocinctus* within *Hydrophis* (Figure 4) was also found by a more comprehensive molecular phylogeny [46].

**Figure 5:**
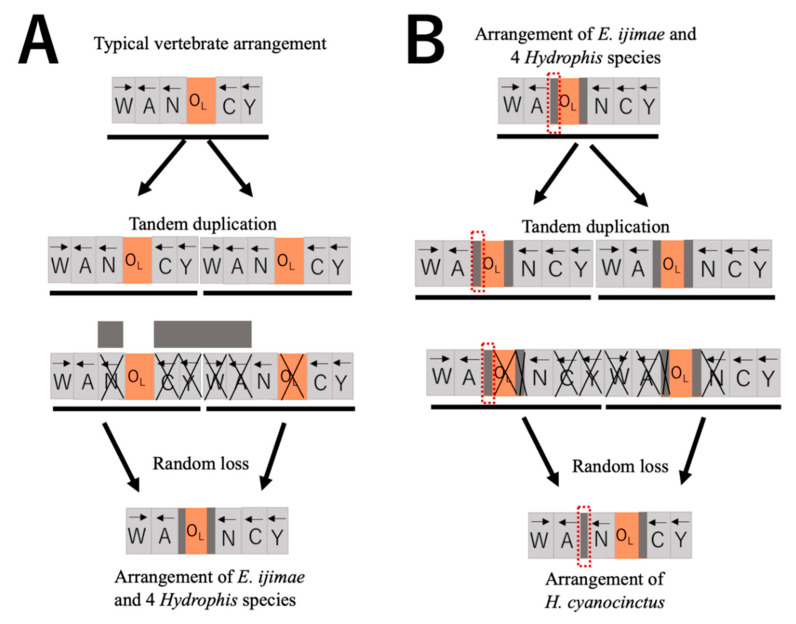
Plausible pathways for the O_L_ translocation (**A**) and its reversal (**B**) by the Tandem Duplication—Random Loss model [47]. X shows genes and O_L_ randomly chosen to be deleted by the model. Gray boxes show intergenic spacer sequences and an intergenic region circled by red dots includes nucleotide sequences with sequence similarity among all 5 *Hydrophis* species and *E. ijimae* (Figure S1).

Various examples of mtDNA gene rearrangements have been known for vertebrates and many of them are local changes of gene orders by retaining encoded strands for the genes [3]. These gene rearrangements are usually explained by the Tandem Duplication—Random Loss (TDRL) model [47]. The transposition of O_L_ and its reversal may also be explained by the TDRL model (Figure 5). After the entire WANCY region is tandemly duplicated, tRNA genes and O_L_ noted with X in Figure 5A are randomly chosen for deletion, resulting in WANCY with the rearranged O_L_ position in it. Here, the spacers between tRNA^Ala^ gene and the O_L_, as well as between the O_L_ and tRNA^Asn^ gene can be interpreted as remnants of deleted regions though we could not detect sequence similarity between the spacers and any of WANCY tRNA genes by BLAST search (data not shown). If WANCY with the transposed O_L_ tandemly duplicated and tRNA genes and O_L_ with the X marking are deleted, WANCY with the reverted O_L_ position arises (Figure 5B). The spacer between tRNA^Ala^ and tRNA^Asn^ genes of *H. cyanocinctus*, which retains sequence similarity to counterparts of *Emydocephalus* and other *Hydrophis* species (Figure S1), provides strong evidence for the transposed O_L_ position in an ancestor of *H. cyanocinctus* and subsequent reversal to the typical position.

### 4.3. Insertion of Long Repetitive Sequences

Between tRNA^Cys^ and tRNA^Tyr^ genes in the WANCY tRNA gene cluster of *S. japonicus* (Elapinae), there was an insertion of long tandem repeats in which a 112-bp unit repeated 32 times (Figure 2; Table S6). Similar insertion of long tandem repeats between tRNA^Cys^ and tRNA^Tyr^ genes, in which a 188-bp unit repeated 10 times, was also found for a congeneric species *Sinomicrurus peinani* (Accession No. MZ230594). However, another congeneric species *Sinomicrurus macclellandi* (Accession No. MT547176) did not have any tandem repeats between tRNA^Cys^ and tRNA^Tyr^ genes.

The slipped-strand mispairing of a nascent DNA strand to repetitive sequences in a template strand during replication [48] is generally considered to be responsible for increasing and diminishing tandem repeats. Based on the ML tree, two equally parsimonious explanations may be possible for the timing of insertion/deletion of the long tandem repeats (Figure 4). One is the insertion of tandem repeats at the common ancestor of *S. japonicus*, *S. peinani* and *S. macclellandi* and subsequent deletion on a lineage leading to *S. macclellandi*. The other is independent insertions of long tandem repeats on lineages leading to *S. japonicus* and *S. peinani* (i.e., branches E and F of Figure 4, respectively). Because the repetitive units for *S. japonicus* and *S. peinani* had distinct sizes and did not show sequence similarity to each other, we tentatively consider that the latter explanation is more likely. It should be noted that mtDNA of the colubrid *Sibon nebulatus* (Accession No. EU728583) also has long tandem repeats between tRNA^Cys^ and tRNA^Tyr^ genes (23 times repetition of a 292-bp unit with no sequence similarity to the repeating units for *S. japonicus* and *S. peinani*). This may also represent parallel insertion of long tandem repeats in elapid (*Sinomicrurus*) and colubrid (*Sibon*) lineages. However, why the long insertion tends to occur at this gene boundary repeatedly remains an open evolutionary question.

### 4.4. Loss of Duplicate Control Regions

The present study revealed that MNCR2 regions of *H. ornatus* and *H. melanocephalus* do not contain any conserved blocks characteristic of the control region, such as CSBs and coreTAS (Figure 3). The MNCR2 of *H. ornatus* (1692 bp) actually consists of three tandem repeats of a 638-bp unit containing the tRNA^Phe^ gene and a 5′ part of the 12S rRNA gene. The MNCR2 of *H. melanocephalus* (2727 bp) also consists of five tandem repeats of a 592-bp unit containing a half of the tRNA^Pro^ gene (pseudogene), the tRNA^Phe^ gene, and a 5′ part of the 12S rRNA gene. Therefore, these MNCR2 regions were simply created by tandem duplication by a mechanism such as the slipped-strand mispairing [48].

The control regions of other snakes (e.g., the elapid *S. japonicus*, the colubrid *L. semicarinatus*, the viperid *O. okinavensis*, and the pythonid *P. regius*) retain high sequence similarities to each other spanning the entire control region of nearly 1 kbp (Figures S2 and S3). We thus conclude that the MNCR2 regions of *H. ornatus* and *H. melanocephalus* lost the control region. We emphasize that this conclusion could be reached only by sequencing the entire MNCR regions accurately. Based on the maximum parsimony criterion, the loss of the control region from MNCR2 likely occurred on a branch C of Figure 4. For the reasons described above, we question the accuracy in sequencing highly repetitive MNCR regions of sea snake mtDNAs by previous investigators. However, reported sequences for MNCR2 regions of *H. cyanocinctus*, *H. platurus*, and *H. curtus* do not contain any evidence for the presence of the control region (Figure S3; Table S7), and this is consistent with our conclusion.

On the other hand, the MNCR1 region was shown to contain the control region in almost all mtDNA sequences determined by us and by previous investigators (Figure 3 and Figure S2; Table S7). The only exception is the mtDNA of *Laticauda semifasciata* (Accession No. KY496325) [40], in which only 259 bp was reported to reside in the MNCR1. This species appears to have the control region at MNCR2 (Table S7). We withhold a conclusion of whether *L. semifasciata* lost the control region from MNCR1 until MNCRs are sequenced accurately from additional *L. semifasciata* individuals in future. The present study also found that *H. melanocephalus* has tandemly duplicated control regions within MNCR1 (Figure 3). Because these duplicate control regions (942bp for each) are identical in sequence, both of them are likely to be functional for regulating the replication and transcription of the mtDNA. This duplication of the control region within MNCR1 was found only for *H. melanocephalus* and it was estimated to have occurred on a branch D of Figure 4 leading to *H. melanocephalus.*

Why have the control regions been stably duplicated in both MNCR1 and MNCR2 for >70 million years of alethinophidian evolution? Why could the control region nevertheless disappear from MNCR2 of *H. ornatus* and *H. melanocephalus*? Can tandemly duplicated control regions within *H. melanocephalus* MNCR1 compensate for its loss from MNCR2? The control region serves as a regulatory region for the H-strand replication and the transcription from both strands [1,4]. Snake mtDNAs with duplicate control regions in MNCR1 and MNCR2 may have acquired ability to enhance the replication and transcription from dual start sites or selectively up-regulate (or down-regulate) expression of genes next to either of the duplicate control regions. To the best of our knowledge, no experimental evidence has been available as to how much each of the duplicate control regions of snake mtDNAs actually contributes to the replication/transcription, except for some bioinformatic inference [49].

Since the duplicate state of the control region in MNCR1 and MNCR2 has persisted in snake mtDNAs for more than 70 million years with continuous concerted sequence evolution between them, this duplicate state of the control region has presumably been subject to positive Darwinian selection [12,13]. Otherwise, this redundant sequence may have been rapidly excised from the mtDNA. Our finding of the snake mitochondrial genomes that may have lost one of the duplicate control regions should provide good study objects to reevaluate such evolutionary hypothesis.

## Figures and Tables

**Table 1:** Elapid species for which mtDNAs were sequenced in this study.

Scientific Name	Classification	Museum Voucher No.	mtDNA Accession No.	mtDNA Length (bp)	MNCR1 Length (bp)	MNCR2 Length (bp)
*Emydocephalus ijimae*	Hydrophiinae; Hydrophiini	KUZ28042	LC648431	20,598	2783	2586
*Hydrophis ornatus*	Hydrophiinae; Hydrophiini	KUZ21782	LC648430	19,682	2733	1692
*Hydrophis melanocephalus*	Hydrophiinae; Hydrophiini	NUM-A0368	LC648429	26,316	8330	2727
*Sinomicrurus japonicus*	Elapinae; Calliophini	SDNCU-A4882	LC648432	21,581	1028	1008

KUZ: Kyoto University Museum (Zoological Collection); NUM: Nagoya University Museum; and SDNCU: Specimen Depository of Graduate School of Science, Nagoya City University.

## Data Availability

The complete mtDNA sequences newly reported in this work have been deposited to the DDBJ database under accession numbers LC648429–LC648432.

## Supplementary Materials

The following supporting information can be downloaded at: www.mdpi.com/article/10.3390/genes13081470/s1, Figure S1: Sequence comparison of the WANCY tRNA gene cluster region among 21 snake species based on clover-leaf secondary structures of tRNA genes; Figure S2: Sequence comparison of the MNCR1 region among 21 snake species; Figure S3: Sequence comparison of the MNCR2 region among 21 snake species; Figure S4: Sequence comparison of tRNA^Ile^ genes among 21 snake species based on their clover-leaf secondary structures; Figure S5: Sequence comparison of tRNA^Phe^ genes among 21 snake species based on their clover-leaf secondary structures; Figure S6: Maximum likelihood tree using nucleotide sequences of all 37 mitochondrial genes; Figure S7: Maximum likelihood tree using amino acid sequences of 13 mitochondrial protein genes; Table S1: PCR primers used to amplify MNCR regions and Nanopore sequence reads obtained; Table S2: Long PCR primers used to amplify mtDNA regions for Illumina sequencing; Table S3: Annotation of the mtDNA sequence of *Emydocephalus ijimae*; Table S4: Annotation of the mtDNA sequence of *Hydrophis ornatus*; Table S5: Annotation of the mtDNA sequence of *Hydrophis melanocephalus*; Table S6: Annotation of the mtDNA sequence of *Sinomicrurus japonicus*; Table S7: Comparison of two major noncoding regions between different species and different INSD sequence entries of sea snakes; Table S8: Twenty one snake species for which mtDNA sequences were compared in this study.
