# Supplementary material for: Evolution of the Noncoding Features of Sea Snake Mitochondrial Genomes within Elapidae
Source: Genes (Basel). 2022 Aug 17;13(8):1470. doi: 10.3390/genes13081470 (PMC9407768; doi:10.3390/genes13081470)
Supplement: Supplementary file 1 [file genes-13-01470-s001.zip › supplementary figure.pdf]

```
  scientific name              Accession number  [→                                    tRNA-Trp                                    →]     [←
 1.Emydocephalus ijimae         LC648431         AGGAACT-TA-AGTT-ATAACACA-AACT-A-GAGGC-CTTCA-AA-GCCAC--CAAA--AAAG-ATCA----CTTT-AGTTCCTG--C----TAGAGTCT-GCGGC-
 2.Hydrophis ornatus            LC648430         AGGAACT-TA-AGTT-ATAATATA-AACT-A-GAGGC-CTTCA-AA-GCCCC--CAAA--AAAG-ATC-----ATTT-AGTTCCTG--C----CAGAATCT-GCGGC-
 3.Hydrophis melanocephalus     LC648429         AGGAACT-TA-AGTT-ATATTGCA-AACT-A-GAGGC-CTTCA-AA-GCCCC--CAAA--AAAG-ACCA----TTTT-AGTTCCTG--C----CAGAGTCT-GCGGC-
 4.Hydrophis curtus             MK953549         AGGAACT-TA-AGTT-ATACTGCA-AACT-A-GAGGC-CTTCA-AA-GCCCC--CAAA--AAAG-ACCA----TTTT-AGTTCCTG--C----CAGAGTCT-GCGGC-
 5.Hydrophis platurus           MK775530         AGGAACT-TA-AGTT-ATATAGCA-AACT-A-GAGGC-CTTCA-AA-GCCCC-CAAAA--AAAG-ACCA----TTTT-AGTTCCTG--C----CAGAGTCT-GCGGC-
 6.Hydrophis cyanocinctus       NC046795         AGGAACT-TA-AGTT-ATATTGCA-AACT-A-GAGGC-CTTCA-AA-GCCCC-CAAAAA-AAAG-ACCA----TTTT-AGTTCCTG--C----CAGAGTCT-GCGGC-
 7.Laticauda semifasciata       KY496325         AGGAACT-TA-AGTT-ATATTA---AACT-A-GAGGC-CTTCA-AA-GCCCC--CAAA--AAAG-ACTA----CTTT-AGTTTCTG--AC---TAGAATCT-GCAGA-
 8.Laticauda colubrina          KY496324         AGAAACT-TA-AGTT-ATATTA---AACT-A-GAGGC-CTTCA-AA-GCCCC--CAAA--AAAG-ACCA----CTTT-AGTTTCTG--CC---TAGAATCT-GCAGA-
 9.Laticauda laticaudata        KY496323         AGAAACT-TA-AATT-ATACA----AACT-A-GAGGC-CTTCATAA-GCCCC--CAAA--AAAG-ACTA----CTTC-AGTTTCTG--AT---TAGAACCT-GAAGA-
10.Sinomicrurus japonicus       LC648432         AGGAACT-TA-AGTT-ATACTAA--AACT-A-GGGGC-CTTCA-AA-GCCCT--CAAA--AAAG-ACA-----ATTT-AGTTCCTG--CCC--TAGAGTCT-GCGGC-
11.Sinomicrurus peinani         MZ230594         AGGAACT-TA-AGTT-ATATA----AACT-A-GAGGC-CTTCA-AA-GCCCC--CAAA--AAAG-AC------ATTT-AGTTCCTG--CC---TAGAGTCT-GCGGC-
12.Sinomicrurus mecclellandi    MT547176         AGGAACT-TA-AGTT-ATACA----AACT-A-GAGGC-CTTCA-AA-GCCCC--CAAA--AAAG-AC------ATTT-AGTTCCTG--CC---TAGAGTCT-GCGGC-
13.Micrurus fulvius             GU045453         AGGAACT-TA-AGTT-ATACTA---AACT-A-GAGGC-CTTCA-AA-GCCCC--CAAA--AAAG-ACCA----CTTT-AGTTTCTG--C----TAGAGCCT-GCGGC-
14.Ophiophagus hannah           EU921899         AGGAACT-TA-AGTT-ATACTA---AACT-A-GAGGC-CTTCA-AA-GCCCC--AAAA--AAAG-ACCA----CTTT-AGTTCCTG--CC---TAGAGCCT-GCGGT-
15.Naja atra                    EU913475         AGGAACT-TA-AGTT-ATACTA---AACT-A-GAGGC-CTTCA-AA-GCCCC--CAAA--AAAG-ACCA----CTTT-AGTTTCTG--TC---TAGAGCCT-GCGGC-
16.Bungarus multicinctus        EU579522         AGAAACT-TA-AGTT-ATATTTA--AACT-A-GAGGC-CTTCA-AA-GCCCC--AAAA--AAAG-ACTA----CTTT-AGTTTCTG--CT---TAGAGCCT-GCAGC-
17.Bungarus fasciatus           EU579523         AGGAACT-TA-AGTT-ATAATA---AACT-A-GAGGC-CTTCA-AA-GCCCC--AAAA--AAAG-ACCA----CTTT-AGTTCCTG--CC---CAGAGCCT-GCAGC-
18.Lycodon semicarrinatus       AB008539         AGAAGCT-TA-AGTT-ATATTA---AACT-A-GGAAC-CTTCA-AA-GTCCC--AAAA--AAAG-ACCA----CTTT-AGTTTCTG--TT---TAGAGCTT-GCAGA-
19.Sibon nebulatus              NC013985         AGGAACT-TA-AGTT-ATACTTA--AACT-A-GGGGC-CTTCA-AA-GCCCC--CAAA--AAAA-CCA-----CTTT-AGTTCCTG--T----TAGAACTT-GCGAA-
20.Ovophis okinavensis          AB175670         AGAAACT-TA-AGTT-ATATTA---AACT-A-GGAGC-CTTCA-AA-GCCCC--AAAT--AAAG-CGCA----CTTT-AGTTTCTG-------CAGAGCTT-GCAGC-
21.Python regius                AB177878         AGAAACT-TA-AGTT-ATATCA---AACT-A-GGAGC-CTTCA-AA-GCCCC--CAAA--AAAG-AAACACA-CTTT-AGTTTCTG-------GAGAACTT-GCGAA-
```

```
                     tRNA-Ala                            ←]                                              [               L-origin               ]
-TAC-ACCAC-ATCA-CCTGC-TTGCAAC-ACAGA-T-ATTT--TACCTT-AAAC-TA-AGACTCC--AGCCCACAAACAAACCTCGCCCAAGATGCCGGACATCAACCCAGC--TTTCTCCGTTTTT-ATTAACGGCGGGGA-AAAAACGGAGAAA--CCCCGGGC
-TAC-ACCAC-ATCA-CCTGA-TTGCAAT-ACAGG-A-GTTT-TTACCTT-AAAC-TA-AGACTCT------------------AACCCAATATGACGGGCAT-AACCCAGC--TTTCTCCGTTTTT-----GGGCGGAAGA-AAAAACGGAGAAA--CCCCGGGC
-TAC-ACCAC-ATCA-CCTGG-TTGCAAC-ACAGG-G-ATTT-TTACCTT-AAAC-TA-AGACTCT------------------AACCCAATATAACGGGCAT-AACCCAGC---TTCTCCGTTTTT-----GGAGGGGGGA-AAAAACGGAGAA---CCCCGGGC
-TAC-ACCAC-ATCC-CCTGA-TTGCAAC-ACAGG-G-ATTT-TTACCTT-AAAC-TA-AGACTCT------------------GCCCCAATATGACGGACAT-ACCCCAGC--TTTCTCCGTTTT------GGGGGGGTG--AAAACGGAGAAA--CCCCGGGC
-TAC-ACCAC-ATCA-CCTGA-TTGCAAT-ACAGG-A-ATTT-TCACCTT-AAAC-TA-AGACTCC------------------AGCCCAATATGGCGAACATTAACCCAGC--TTTCTCCGTTTTT----TGACGGAGAAG-AAAAACGGAGAAA--CCCCGGGC
-TAC-ACCAC-ATCA-CCTGG-TTGCAAC-ACAGG-G-ATTT-TTACCTT-AAAC-TA-AGACTCT------------------AACCCAATATAACGGGCAT-AACCCAGC---------------------------------------------------
-TA--ACCAC-ATCA-CCTGA-CTGCAAT-ACAGG-T-ATTT--TAACTT-AAAC-TA-AGACTCT------------------------------------------------------------------------------------------------
-CAC-GCCAC-ATTG-CCTGA-TTGCAAT-ACAGG-A-GTTT--TAACTT-AAAC-TA-AGATTCC------------------------------------------------------------------------------------------------
-CTG-ACCAT-ATCA-CCTAA-TTGCAAT-ACAGG-A-GTTT--TAGCTT-AAAC-CA-GGACTCC------------------------------------------------------------------------------------------------
-TAC-ACCAC-ATCA-CCTGC-TTGCAAC-ACAGG-T-ATTT--AAACTT-AAAC-TA-AGACTCC------------------------------------------------------------------------------------------------
-TAA-ACCAC-ATCA-CCTGT-TTGCAAA-ACAGA-T-ATTT--CAAATT-AAAC-TA-AGACTCC------------------------------------------------------------------------------------------------
-TAA-ACCAC-ATCA-TCTGT-TTGCAAT-ACAGA-T-ATTT--CAAAT--AAAC-TA-AGACTCC------------------------------------------------------------------------------------------------
-TTC-ACCAC-ATCA-CCTGC-TTGCAAC-ACAGA-T-ATTT--TAACTT-AAAC-TA-AGACTCT------------------------------------------------------------------------------------------------
-TAC-ACCAC-ATCA-CCTGC-CTGCAAC-ACAGA-T-ATTT--TAATT--AAAC-TA-AGACTCC------------------------------------------------------------------------------------------------
-TAC-ACCAC-ATCA-TCTGA-CTGCAAC-TCAGA-T-ATTT--TATCTT-AAAC-TA-AGGCTCT------------------------------------------------------------------------------------------------
-TAC-ACCGC-ATCA-CCTAT-ATGCAAC-ATAGA-T-ATTT--TAACTT-AAAC-TA-AGACTCC------------------------------------------------------------------------------------------------
-AAC-ACTAC-ATCA-TTTAT-ATGCAAC-ATAAA-T-ATTT--TACTT--AAAC-TA-AGACTCC------------------------------------------------------------------------------------------------
-AAC-ACCAC-ATCT-ACTGC-TTGCAAC-ACAGA-C-ATTT--TAATT--AAAC-TA-AAGCTCT------------------------------------------------------------------------------------------------
-C---ATCAC-ATCT-CCTGA-TTGCAAC-ACAGA-C-ATTT--TAATT--AAAC-TA-AAGCTCT------------------------------------------------------------------------------------------------
-ATC-ACCAC-ATCT-CCTGC-TTGCAAC-ACAGA-C-ATTT--TAACT--AAAC-TA-AAGCTCC------------------------------------------------------------------------------------------------
-CA--ATCAC-ATCT-TCTGC-TTGCAAC-ACAGA-T-ATTT--TATCT--AAAC-TA-AAGCTCT------------------------------------------------------------------------------------------------
```

```
                                                                            [←                                   tRNA-Asn                                  ←
AGGAAAGCTGATTACCATCTTTGCAGAATCA--------------------------------------------CTAGGTTA-GTGGG-CCTCGAT-CCCAC-AAATA-ACTAA-TTAACAG-TTAGC-T-GTCT-AAGCCGGC--TGAC-AT-TAACCTA
A--AAAGCTGGAGGCAGGGGGTTTACGCCCTGACCTAATATGACCGGCCAGTGTATAAAATAACACGCTTTTATGTC--CTGAGTCA-GCGGG-CTTTGAT-CCCGC-AAATA-ACTAA-TTAACAA-TTAGC-T-GTCC-AAACCAAC--GGAC-CT-TGGCCCA
AG--AAGCTGGAGGCAGATGATT-ACGCCTTGACCTAATATGACCGACCGGTGTATAAAATA-CAC----TTGTGTC--CTGAGTCA-GTGGG-CTTTGAT-CCCAC-AAATA-ACTAA-TTAACAA-TTAGC-T-GCCC-AAACCAAC--GGAC-AT-TGACCCA
---------------------------------------------------------------------------CTGAGTCA-GCGGG-CTTTGAT-CCCAC-AAATA-ACTAA-TTAACAA-TTAGC-T-GTCC-TAACCGAC--GGAC-AT-TGACCCA
AG-AAAGCTGGAGGCAGGTGTGGTACGCCATGACCTATTATGACCGGCCGGTG-ATAAA-TACCAC-TACCTGTGCC--CTGAGTCA-GTGGG-CTTTGAT-CCCAC-AAATA-ACTAA-TTAACAA-TTAGC-T-GCCC-AAACCAAC--GGAC-GT-TGACCCA
---------------------------------------------------------------------------CTGAGTCA-GTGGG-CTTTGAT-CCCAC-AAATA-ACTAA-TTAACAA-TTAGC-T-GCCC-AAACCAAC--GGAC-AT-TGACCCA
---------------------------------------------------------------------------CTAGATCA-GTGGG-CCTTGAT-CCCAC-AAATA-ACTAA-TTAACAG-CTAGT-C-GTCC-AAACCGGC--GGAC-TT-TAACCTA
---------------------------------------------------------------------------CTAGGTCA-GGGGG-CCTCGAT-CCCAC-AAACA-ACTAA-TTAACAG-CTAGT-C-ATCC-AAACCGGC--GGAC-TT-TAACCTA
---------------------------------------------------------------------------CTAGGTCA-GCGGA-CCTTGAT-CCCAC-AAACA-ACTAA--TAACAA-CAAGT-C-ATCC-AATCCGAC--GGAC-TT-TAACCTA
---------------------------------------------------------------------------CTAGATTA-GTGGG-CCTCGAT-CCCAC-AAACA-ACTAA-TTAACAG-CTAGC-T-GTCC-AAACCGGC--GGAC-CT-TAACCTA
---------------------------------------------------------------------------CTAGATTA-GTGGG-CCTCGAT-CCCAC-AAATA-ACTAA-TTAACAG-TTAGC-T-GTCC-AAGCCGGC--GGAC-CT-TAACCTA
---------------------------------------------------------------------------CTAGATTA-GTGGG-CCTCGAT-CCCAC-AAACA-ACTAA-TTAACAG-TTAGC-C-GTCC-AAACCGAC--GGAC-AT-TAACCTA
---------------------------------------------------------------------------CTAGATTA-GCGGG-CCTCGAT-CCCAC-AAATA-ACTAA-TTAACAG-TTAGC-T-GTCC-AAACCGAC--GGAC-TT-TAACCTA
---------------------------------------------------------------------------CTAGATTA-GTGGG-CCTCGAT-CCCAC-AAACA-ACTAA-TTAACAG-CTAGC-T-GTCC-AAACCGGC--GGAC-CT-TAACCTA
------------------------------------------------------------------------T--CTAGATCA-GTGGG-CCTTGAT-CCCAC-AAACA-ACTAA-TTAACAG-TTAGT-C-GTCC-AAACCGGC--GGAC-TT-TAACCTA
---------------------------------------------------------------------------CTAGATTA-GTGGG-CCTCGAT-CCCAC-AAACA-ACTAA-TTAACAG-CTAGC-T-ATCC-AAACCGGC--GGAC-CT-TAATCTA
---------------------------------------------------------------------------CTAGATTA-GTGGG-CCTTGAT-CCCAC-AAACA-ACTAA-TTAACAG-CTAGC-T-ATCC-AAACCGGC--GGAC-CT-TAATCTA
---------------------------------------------------------------------------CTAGATTA-GCGGG-CCTCGAT-CCCAC-AAAA--ACTAA-TTAACAG-CTAGC-T-GTCA-AAACCGGC--GGAC-TT-TAATCTA
---------------------------------------------------------------------------CTAGACTA-GCGGG-CCTCGAT-CCCAC-AAAA--ACTAA-TTAACAG-CTAGC-T-GTCC-AAACCGGC--GGAC-TT-TAATCTA
---------------------------------------------------------------------------CTAGATTA-GCAGG-CCTCGAT-CCTAC-AAAAA-ACTAA-TTAACAG-TTAGC-C-GTCC-AAACCGTC--GGAC-TT-TAACCTA
---------------------------------------------------------------------------CTAGACTG-GCGGG-CCTCGAT-CCCAC-AAAAC-ACTAA-TTAACAA-CTAGC-C-GTCC-AAACCAAAC-GGAC-TT-CAATCTA
```

```
-]              [                  L-origin                  ]                                                         [←                  tRNA-Cys
--TGGG-------------------------------------------------------------------------------------------------------GAAACCCT-TAGCA--AAT-GCGCC-TACT-TTAGA-TTTGCAG-TCTAA-C-
--CCACTATATAAAT--------------------------------------------------------------------------------------------AAAACTCC-TGGCA--GA--TAGCC-TACT-TCAGA-TTTGCAC-TCTGA-T-
--CCATATATAAAT---------------------------------------------------------------------------------------------AAAACTCC-TGGCA--AA--TAGCC-TACT-TCAGA-TTTGCAC-TCTGA-T-
--CCATTGCATAAA---------------------------------------------------------------------------------------------TAAACCCC-TGGCA--GA--TAGCC-TACT-TCAGA-TTTGCAC-TCTGA-T-
--CCATTATATAAAT--------------------------------------------------------------------------------------------TAAACTCC-TGGCA--GA--TAGCC-TACT-TCAGA-TTTGCAC-TCTGA-T-
------------------TTCTCCGTTTTT---GGAGGGGGGGA---AAAAACGGAGAA---MCMMKKRSMKRMSCWRRCCAACGGACATTGACCCACCATGTATAAAT--AAAACTCC-TGGCA--AA--TAGCC-TACT-TCAGA-TTTGCAC-TCTGA-T-
--GC-------------TTTCTCCGTTTTT---GTAATAGAAGAAA-AAAAACGGAGAAA-----------------------------------------------------CCCC-GGGCA--GC--TAGCC-GACT-TCAGA-TTTGCAG-TCTGA-C-
--GT-------------TTTCTCCGTTTTT---GTAAGGAAAGAA--AAAAACGGAGAAA-----------------------------------------------------CCCC-GGGCA--AAC-TAGCC-GACT-TCAGA-TTTGCAG-TCTGA-C-
--GC-------------TTTCTCCGTTTTT-TGGTGGGGAATAAA--AAAAACGGAGAAA-----------------------------------------------------CCCC-GGGCA--CC--CAGCC-AACT-TCAGA-TTTGCAG-TCTGA-C-
--GC-------------TTTCTCCGTTTTT---GGGGGGGAGGAA--AAAAACGGAGAAA-----------------------------------------------------CCCC-GGGCA--GA--AAGCC-GACT-TCAGA-TTTGCAG-TCTGA-C-
--GC-------------TTTCTCCGTTTTT---GGAGGGGGGGAA--AAAAACGGAGAAA-----------------------------------------------------CCCC-GGGCA--GA--AAGCC-GACT-TCAGA-TTTGCAG-TCTGA-C-
--GC-------------TTTCTCCGTTTTT---GGGGGGGGGGAA--AAAAACGGAGAAA-----------------------------------------------------CCCC-GGGCA--GA--AAGCC-GACT-TCAGA-TTTGCAG-TCTGA-C-
--GC-------------TTTCTCCGTTTTT---ATTAACGATGGGA-AAAAACGGAGAAA-----------------------------------------------------CCCC-GGGCA--G---AAGCC-TACT-TCAGA-TTTGCAG-TCTGA-C-
--GC-------------TTTCTCCGTTTTT---ATAACGGAAGGA--AAAAACGGAGAAA-----------------------------------------------------CCCC-GGGCA--GC--TAGCC-TACT-TCAGA-TTTGCAG-TCTGA-C-
--GC-------------TTTCTCCGTTTTT---GTAACGGGAAAA--AAAAACGGAGAAA-----------------------------------------------------CCCC-GGGCA--GC--TAGCC-TACT-CCAGA-TTTGCAG-TCTGA-C-
--GC-------------TTTCTCCGTTTT----GTGGGGGGAAA----AAAACGGAGAAA-----------------------------------------------------CCCC-GGGCA--A---AGGCC-GACT-TCAGA-TTTGCAG-TCTGA-C-
--GC-------------TTTCTCCGTTTTT---ATAAAAAGGAAA--AAAAACGGAGAAA-----------------------------------------------------CCCC-GGGCA--GA--AAGCC-GACT-TCAGA-TTTGCAG-TCTGA-C-
--GC--------------TTCTCCGTTTTT---GGACGAAAGA----AAAAACGGAGAA-----------------------------------------------------ACCCG-GGGCC--G---CTGCC-GACT-TCAGA-TTTGCAG-TCTGA-C-
--G--------------CTTCTCCGTTTTT---GAAACGGGAAA---AAAAACGGAGAAG-----------------------------------------------------CCCC-GGGCC--GT--ATGCC-GACT-TCAGA-TTTGCAG-TCTGA-C-
--C--------------CTTCTCCGTTTTT---GGGGGGAAGA----AAAAACGGAGAAG-----------------------------------------------------CCCC-GGGCG--GA--GGGCC-GACT-TCAGA-TTTGCAG-TCTGA-C-
-----------------CTTCTCCGTTTT----AACAAAGAA------AAAACGGAGAAG-----------------------------------------------------CCCC-GGACC--AGT-TGGTC-GTCT-TCGGG-TTTGCAA-CCCGA-C-
```

```
                       ←][→ repeat region
-GTGT-GTT--ACAC-CC-AAGGGTC------------------------------------------------------------------------------------------------------------------------------------
-ATGT-ATT--ACAC-CT-AAGAGTC------------------------------------------------------------------------------------------------------------------------------------
-GTGT-ATT--ACAC-CT-AAGAGTT------------------------------------------------------------------------------------------------------------------------------------
-ATGT-ATT--ACAC-CT-AAGAGTC------------------------------------------------------------------------------------------------------------------------------------
-ATGT-ATT--ACAC-CT-AAGAGTT------------------------------------------------------------------------------------------------------------------------------------
-GTGT-ATT--ACAC-CT-AAGAGTC------------------------------------------------------------------------------------------------------------------------------------
-ATGT-AAAA-ACAC-CT-CAAGGTT------------------------------------------------------------------------------------------------------------------------------------
-ATGT-TAA--ACAC-CT-CAGGGTT------------------------------------------------------------------------------------------------------------------------------------
-ATGT-TAA--ACAC-CT-CAAGGTT------------------------------------------------------------------------------------------------------------------------------------
-ATGT-ATT--ACAC-CT-CGGGGTT--AAACAACGGGCTGAGCCCACCACTATATAGCTACAAACCTCACTACATTTGTAGCGGCTTATATATACTATCACCTCTTAGAAATATACACTTCAGTTGTTACCACACGGGCGAT--[CCTCCATTCTTCATACCTATA
-ATGT-CAAT-ACAC-CT-CGGAGTC--TCGTTAAACTCTTCATGCTAGTTTTAGGTTGCTTACTGTTGAACTCTCCTATTGCATGATCTACTTATTTACCCGCTGCTTGCCTTTAAGTGAGATAGTAGACTTATCGGACCAGCCACAGGTCTTGACTAAGTATTCA
-ATGT-CAC--ACAC-CT-CGGAGTT------------------------------------------------------------------------------------------------------------------------------------
-ATGT-AAC--ACAC-CT-CAAGGTT------------------------------------------------------------------------------------------------------------------------------------
-GTGT-CTCT-ACAC-CT-CGAAGTT------------------------------------------------------------------------------------------------------------------------------------
-ATGT-CAC--ACAC-CG-CAGGGTT------------------------------------------------------------------------------------------------------------------------------------
-ATGT-CAA--ACAC-CT-CGGGGTT------------------------------------------------------------------------------------------------------------------------------------
-ATGT-AAC--ACAC-CT-CGAGGTT------------------------------------------------------------------------------------------------------------------------------------
-ATGT-TAC--ACAC-CT-CCGGGTT------------------------------------------------------------------------------------------------------------------------------------
-ATGA-TAAT-ACAC-CT-CGAAGCT--[CTGGCAGTGGGGTCCGGTCCACCCTATCTGAAATCTGTAAAATTACCGTTTCTATTAACATACTGCCTGTGTTTATCACCGTTGACTCTTCTCAACTGCCGCTTACAAGACCCTAAATTTATACCACGAGCGGATATA
-ATGA-TA---ACAC-CT-CGGGGCC------------------------------------------------------------------------------------------------------------------------------------
-ATGA-A----ACAC-TT-CGGGGCC------------------------------------------------------------------------------------------------------------------------------------
```

```
--------------------------------------------------------------------------------------------------------------------------------------------------------------
--------------------------------------------------------------------------------------------------------------------------------------------------------------
--------------------------------------------------------------------------------------------------------------------------------------------------------------
--------------------------------------------------------------------------------------------------------------------------------------------------------------
--------------------------------------------------------------------------------------------------------------------------------------------------------------
--------------------------------------------------------------------------------------------------------------------------------------------------------------
--------------------------------------------------------------------------------------------------------------------------------------------------------------
--------------------------------------------------------------------------------------------------------------------------------------------------------------
--------------------------------------------------------------------------------------------------------------------------------------------------------------
AAACGCCACTAAAAAATAAAATATATGTTGCTTTTATCACGACCCGCCACAGTCTAATTAAAGCATATTTCCATTACCGTACTACCCATAAA] 32 tandem repeats of 112bp sequence---680bp of repeat-prone sequence----
ATATCATCTACTCAGAACAATAATGTAGGCCTAGTATACACGCTCACCCCTATGGGCAATTAAATACAGGCTTAATTATGACGACTGCGGGTCTTGACTAAAGTAGTCTTATCCCATATAAAACTACTTTATCATTGACCAGCCACAGGTCTTGACTAAACATACA
--------------------------------------------------------------------------------------------------------------------------------------------------------------
--------------------------------------------------------------------------------------------------------------------------------------------------------------
--------------------------------------------------------------------------------------------------------------------------------------------------------------
--------------------------------------------------------------------------------------------------------------------------------------------------------------
--------------------------------------------------------------------------------------------------------------------------------------------------------------
--------------------------------------------------------------------------------------------------------------------------------------------------------------
--------------------------------------------------------------------------------------------------------------------------------------------------------------
AACCTCTGACTTCTGCCATTAGCCCTACTTCTATGATCCTCATACATAGAAACTAGAGCCGGCAGGGACAGAACAATATCCCTCCTCCACCCTGGCAATTTCTCCTTACACTTAGGAAATACGTCCTCCATCCAAGAAGGACCAATTTATTATATAA] 23 tandem
--------------------------------------------------------------------------------------------------------------------------------------------------------------
--------------------------------------------------------------------------------------------------------------------------------------------------------------
```

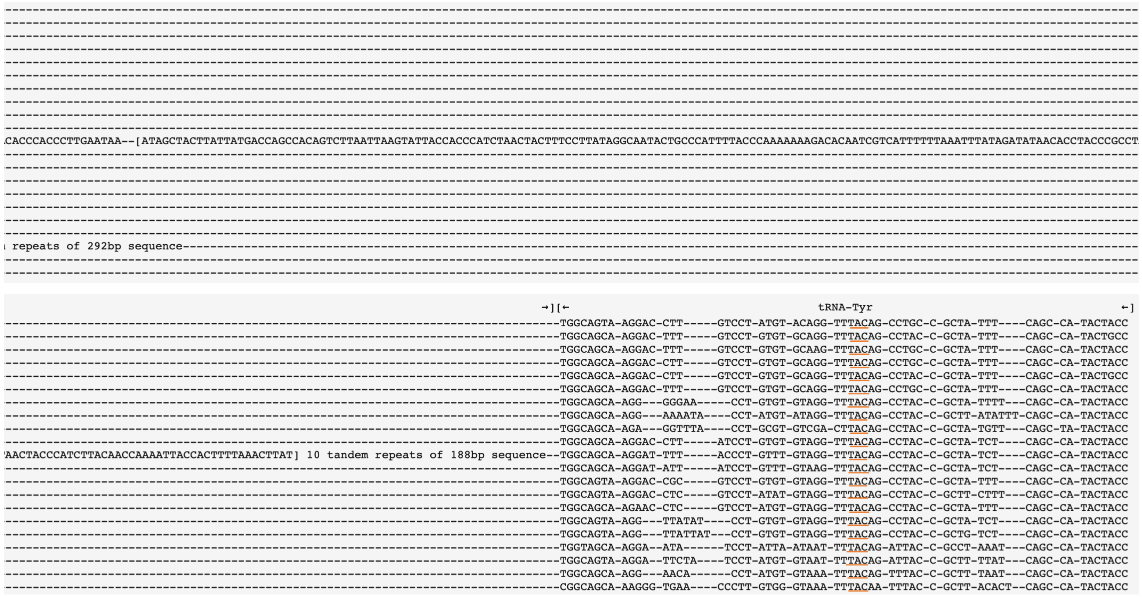

Supplementary figure S1. Sequence comparison of the WANCY tRNA gene cluster region among 21 snake species based on clover-leaf secondary structures of tRNA genes. The red line indicates base-pairing stem region of the L-strand replication origin, as well as the anticodon of each tRNA gene. Note that the first 3-4 bases of the tRNA^{cys} genes for 15 snake species overlap with the L-strand replication origin.

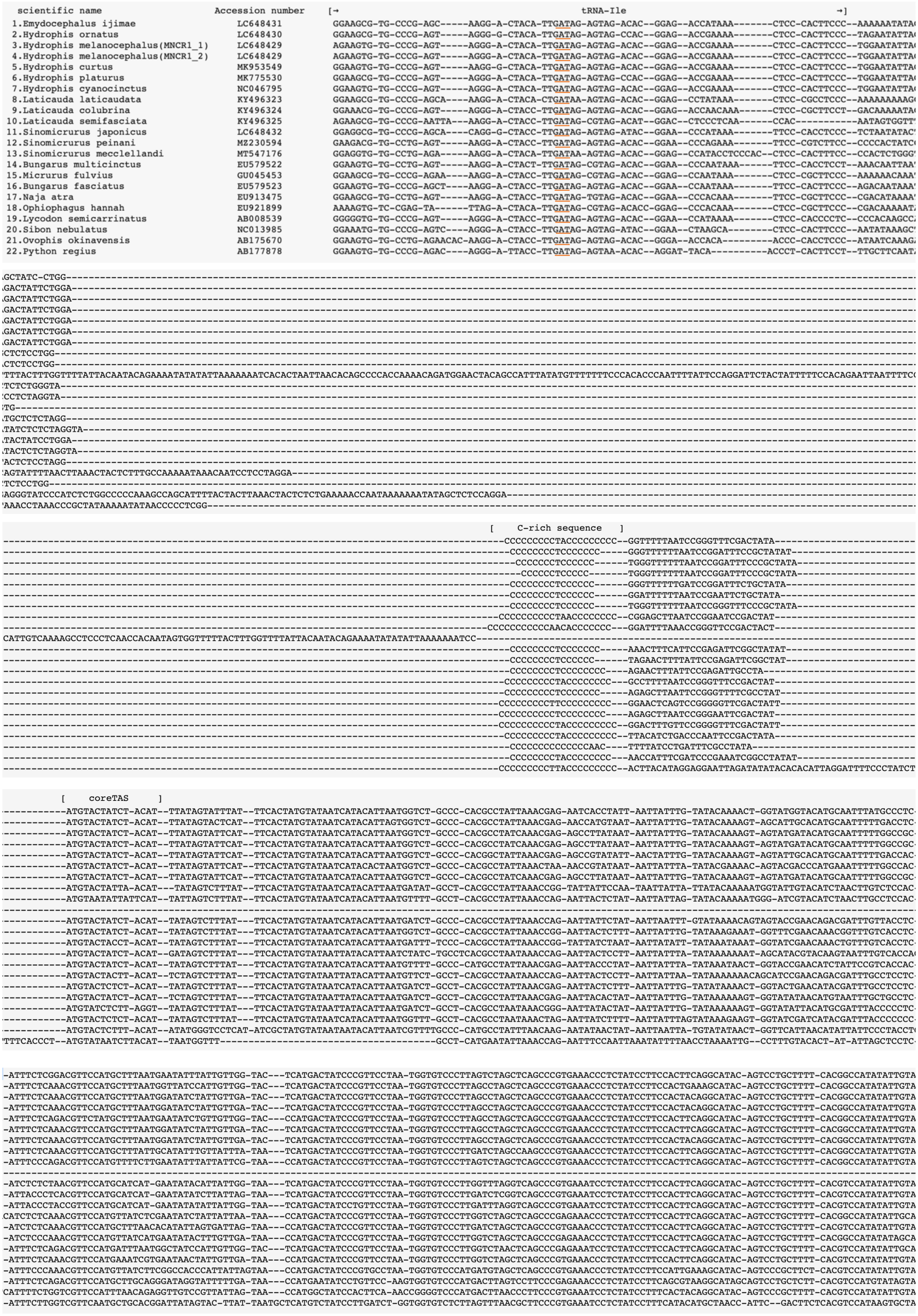
scientific name
Accession number
tRNA-Ile
1.Emydocephalus ijimae LC648431
2.Hydrophis ornatus LC648430
3.Hydrophis melanocephalus(MNCR1_1) LC648429
4.Hydrophis melanocephalus(MNCR1_2) LC648429
5.Hydrophis curtus MK953549
6.Hydrophis platurus MK775530
7.Hydrophis cyanocinctus NC046795
8.Laticauda laticaudata KY496323
9.Laticauda colubrina KY496324
10.Laticauda semifasciata KY496325
11.Sinomicrurus japonicus LC648432
12.Sinomicrurus peinani MZ230594
13.Sinomicrurus mecclellandi MT547176
14.Bungarus multicinctus EU579522
15.Micrurus fulvius GU045453
16.Bungarus fasciatus EU579523
17.Naja atra EU913475
18.Ophiophagus hannah EU921899
19.Lycodon semicarrinatus AB008539
20.Sibon nebulatus NC013985
21.Ovophis okinavensis AB175670
22.Python regius AB177878
C-rich sequence
coreTAS

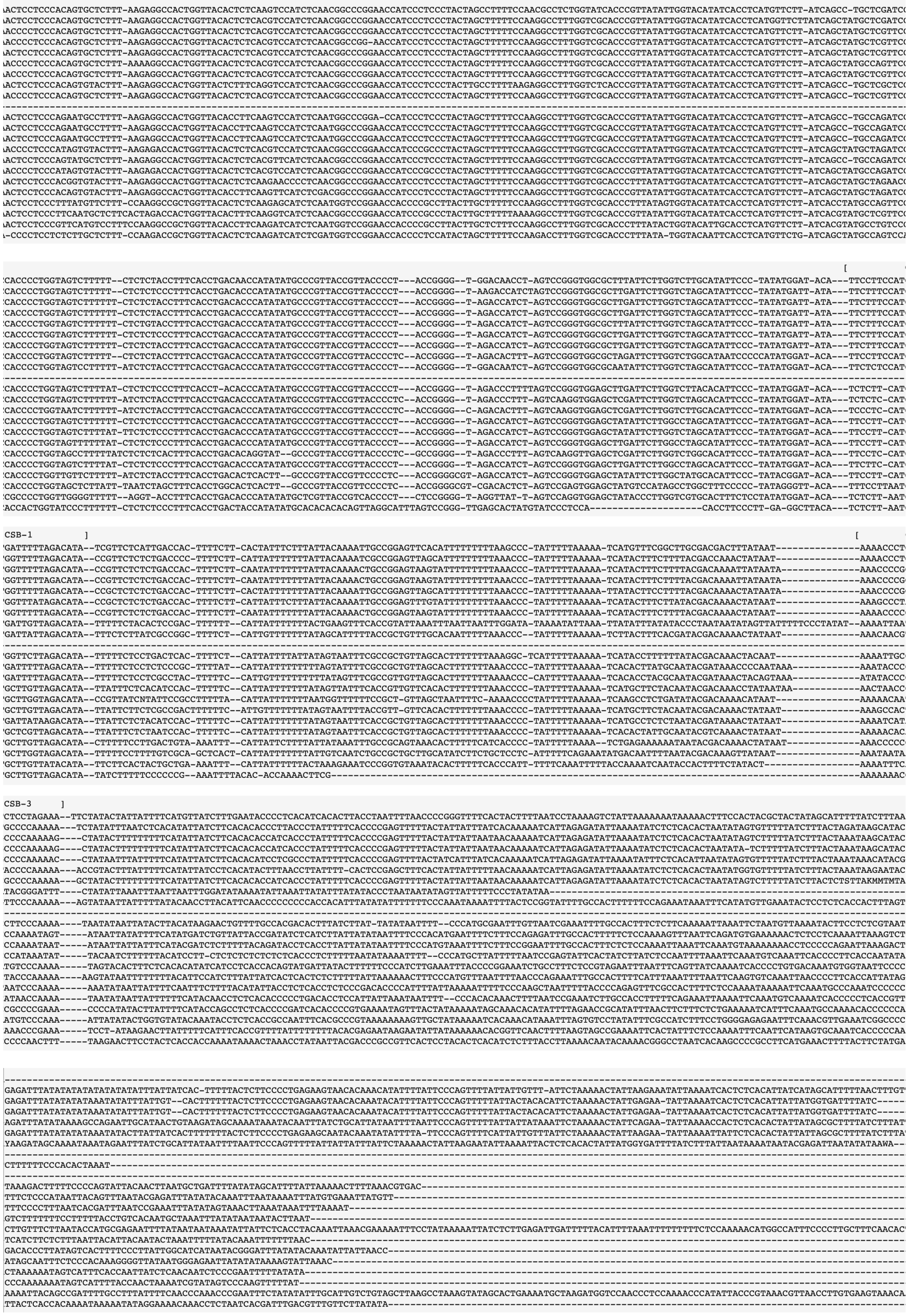
CSB-1
CSB-3

```
--------------------------------------------------------------------------------
TAATAAAATAATACGAGATTAATATATAAA---TATTTTAACCTCACTCAATTTTATTTTTACCCCCTGCATAAGATAGCAAAATAAATACAATTTATCTGCATTATAATTTTAATTCCCTGTTTTTATTATTATTTATTCTAAAAACTATTAAGAATATTA
--------------------------------------------------------------------------------
--------------------------------------------------------------------------------
TAATAAAACAATACGAGATTGATATACAATA-------------------------------------------------
TTAATAAAACAATACGAGATTAATATATAAAAT-----------------------------------------------
--------------------------------------------------------------------------------
--------------------------------------------------------------------------------
--------------------------------------------------------------------------------
--------------------------------------------------------------------------------
--------------------------------------------------------------------------------
--------------------------------------------------------------------------------
--------------------------------------------------------------------------------
--------------------------------------------------------------------------------
TATGCGGAAATTAATAATAACTTCTGAGCGCTATC---------------------------------------------
--------------------------------------------------------------------------------
--------------------------------------------------------------------------------
--------------------------------------------------------------------------------
--------------------------------------------------------------------------------
--------------------------------------------------------------------------------
ACCGATAAATTAAAACTACCCCCACCCATCTTCAACCCATAACCCATGACCCAT--------------------------
--------------------------------------------------------------------------------
```

```
                                                                   [→repeat sequence
-------------------------------------------------------------------[TTATAAAATAATCCGAGATTAATATATAAATATATTCTCCCACCAGCCTCCCTCCCCCAAAAAAAAAGGTACAAGCATGTTTCGTTCGATGT
AAATTATTCTCACGCTATTATAGCTATTTTATCTTACTTATAAAACAATACGAGATTAATATATAA--[AATATTTTAACCTCACTCAATTTTATTTTTACCCCCTGCATAAAGATAGCAAAATAAATACAATTTATCTGCATTATAATTTTAATTCCCTG
-------------------------------------------------------------------[TTTATTAATAAAATAGTACGAGATTAATATATAAAATATTTTAACCTCACTCAATTTTATTTTCA-CCCTTCTACAAGATAGCAAAATAAAT
-------------------------------------------------------------------[TTTATTAATAAAATAGTACGAGATTAATATATAAAATATTTTAACCTCACTCAATTTTATTTTCA-CCCTTCTACAAGATAGCAAAATAAAT
--------------------------------------------------------------------------------
-------------------------------------------------------------------[ATTTTAACCTCACTCAATTTTATTTCAACCCTCTCATTAAGATAGCAAAATAAATATAATTTATCTGCATTATAATTTTTATTCCCAGTTTT
--------------------------------------------------------------------------------
--------------------------------------------------------------------------------
-------------------------------------------------------------------[TACGATACTGATTTTCTATAATAAATCACCGTTCAAAATTTACTCTTGTGTAAAAATACTCCTACTATTTTAATGGTTTTTCCGTATCTATA
--------------------------------------------------------------------------------
--------------------------------------------------------------------------------
--------------------------------------------------------------------------------
--------------------------------------------------------------------------------
--------------------------------------------------------------------------------
--------------------------------------------------------------------------------
--------------------------------------------------------------------------------
--------------------------------------------------------------------------------
--------------------------------------------------------------------------------
--------------------------------------------------------------------------------
--------------------------------------------------------------------------------
--------------------------------------------------------------------------------
--------------------------------------------------------------------------------
```

```
TAATTTTTTATTCCCAGTTTTTACTATTATTAGTTCTAACAAGCCTATTTAAAAATATTAAAATTACTTCTCACACTACTATAGCATTTTTACCTTTAA] 10 tandem repeats of 190bp sequence-----------------------
GTTTTTATTATTATTTATTCTAAAAACTATTAAGAAATATTAAAATCACTCTCACGCTATTATAGCTATTTTATCTTTACTTATAAAACAATACGAGATTAATATATA] 8 tandem repeats of 197bp sequence---------------
TAGAATTTATCTGCATTGTAGCTTTAATTCCCAGTTTTTATTATTAAGTTTCTAAAAACTATTAGGAATATTAAAATTACTCTCGCGCTATTATAGCGATTTTTAT] 13 tandem repeats of 198 bp sequence-------------
TAGAATTTATCTGCATTGTAGCTTTAATTCCCAGTTTTTATTATTAAGTTTCTAAAAACTATTAGGAATATTAAAATTACTCTCGCGCTATTATAGCGATTTTTAT] 14 tandem repeats of 198 bp sequence-------------
--------------------------------------------------------------------------------
TTATTATTGTTTATTCTAAAAACTATTAAGAATATTAAAATTATTCTCACACTATTATTAGCGCTTTTATCTTTATTAATAAAACAATACGAGATTAATATATAAAAT] 4 tandem repeats of 199 bp sequence-------------
--------------------------------------------------------------------------------
--------------------------------------------------------------------------------
ATTACAATACTGATTTTTTATAATAAAAAACTACTATAAAAACCAATAATGACTTAGAATAATTTACGATAAAATAACCACTCCCCCTCAATAATTGTATTAATGCAAAATCTTCCCCACTAATATAGTTATTTTATCCCATGCTTACAG] 2 tandem
--------------------------------------------------------------------------------
--------------------------------------------------------------------------------
--------------------------------------------------------------------------------
--------------------------------------------------------------------------------
--------------------------------------------------------------------------------
--------------------------------------------------------------------------------
--------------------------------------------------------------------------------
--------------------------------------------------------------------------------
--------------------------------------------------------------------------------
--------------------------------------------------------------------------------
--------------------------------------------------------------------------------
--------------------------------------------------------------------------------
--------------------------------------------------------------------------------
```

```
                        →][→                                        tRNA-Phe                                        →]
--------------------------------------------------------------------------------
--------------------------------------------------------------------------------
---------------------------GTTATCA-TA-GCTT-ACAACC---AAGC-A-TAGCA-CTGAAAA--TGCTA-AGAC-GGC-CTT---ACC--TGATAACA--ACCCACAGTGTAATAATGCTATTACAACCCATTATTAAATACAACACTAA
--------------------------------------------------------------------------------
--------------------------------------------------------------------------------
--------------------------------------------------------------------------------
--------------------------------------------------------------------------------
--------------------------------------------------------------------------------
 repeats of 241 bp sequence-----------------------------------------------------
--------------------------------------------------------------------------------
--------------------------------------------------------------------------------
--------------------------------------------------------------------------------
--------------------------------------------------------------------------------
--------------------------------------------------------------------------------
--------------------------------------------------------------------------------
--------------------------------------------------------------------------------
--------------------------------------------------------------------------------
--------------------------------------------------------------------------------
--------------------------------------------------------------------------------
--------------------------------------------------------------------------------
--------------------------------------------------------------------------------
--------------------------------------------------------------------------------
```

```
--------------------------------------------------------------------------------
--------------------------------------------------------------------------------
AAACCGCCCTACTTTATTGAACTAGGTCTCCTATTTACATGGCTACTCAAATATCTTATACATTCATCTCATGATCTAAGCAACCACCAAACCTCTAACAAAGCAATACGCGCCATCATATAGTATTTTATAAAGACCGCACGTAAACCTACTCTTTATAA
--------------------------------------------------------------------------------
--------------------------------------------------------------------------------
--------------------------------------------------------------------------------
--------------------------------------------------------------------------------
--------------------------------------------------------------------------------
--------------------------------------------------------------------------------
--------------------------------------------------------------------------------
--------------------------------------------------------------------------------
--------------------------------------------------------------------------------
--------------------------------------------------------------------------------
--------------------------------------------------------------------------------
--------------------------------------------------------------------------------
--------------------------------------------------------------------------------
--------------------------------------------------------------------------------
--------------------------------------------------------------------------------
--------------------------------------------------------------------------------
--------------------------------------------------------------------------------
--------------------------------------------------------------------------------
```

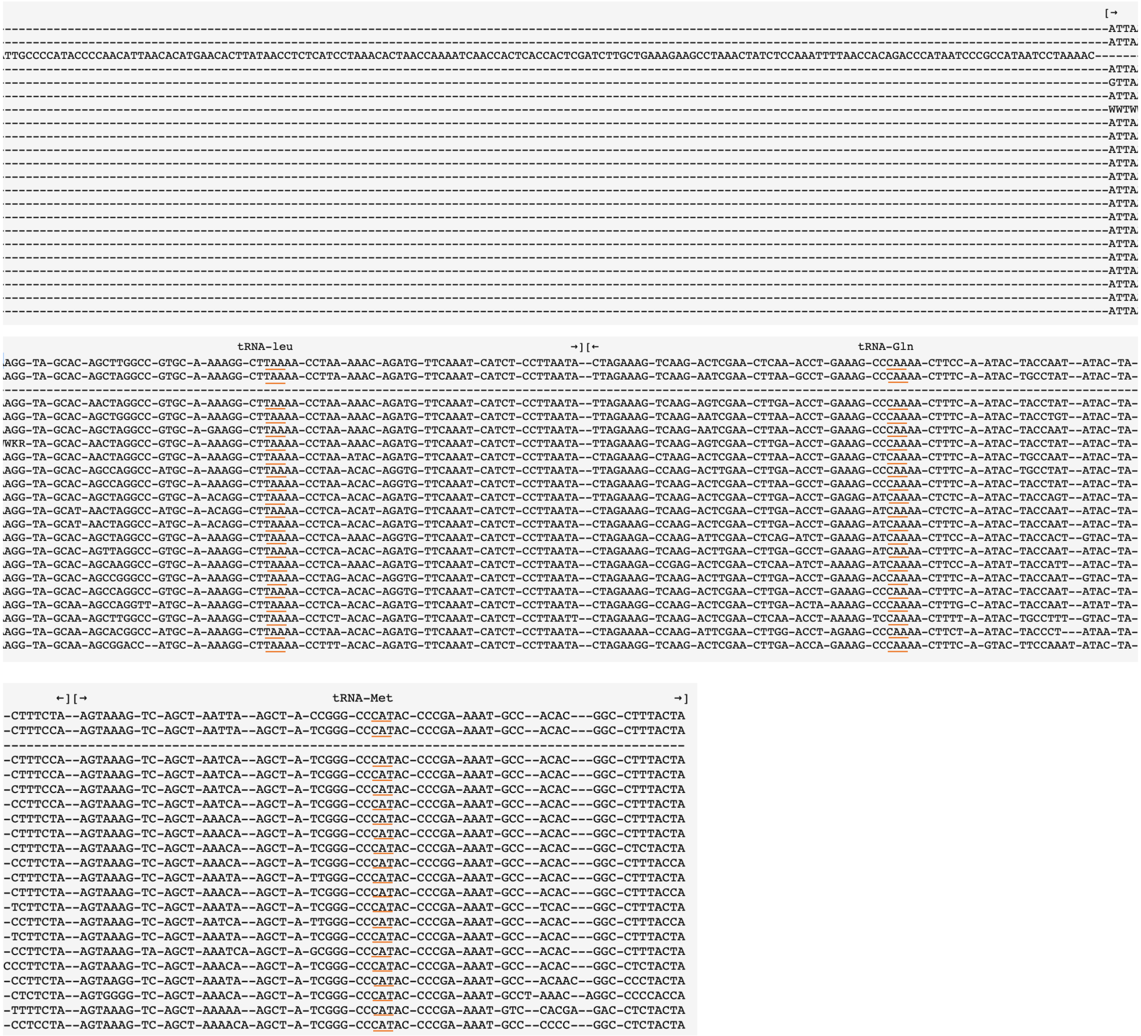

Supplementary figure S2. Sequence comparison of the MNCR1 region among 21 snake species. The control region sequences were aligned with the aid of ClustalX software after identifying common sequence blocks (C-rich, CSB-1, CSB-3, and coreTAS sequences). Transfer RNA genes were aligned based on their clover-leaf secondary structures. The red line indicates the anticodon of each tRNA gene. Sequences for tandemly duplicated regions are represented by a single repetitive unit sequence with a note on repetitive numbers. MNCR1 for *Hydrophis melanocephalus* contains duplicated control regions (see Fig. 3) and they are shown sequentially from MNCR1_1 to MNCR1_2. Note that *Laticauda semifasciata* had a lot of difficulties in the alignment.

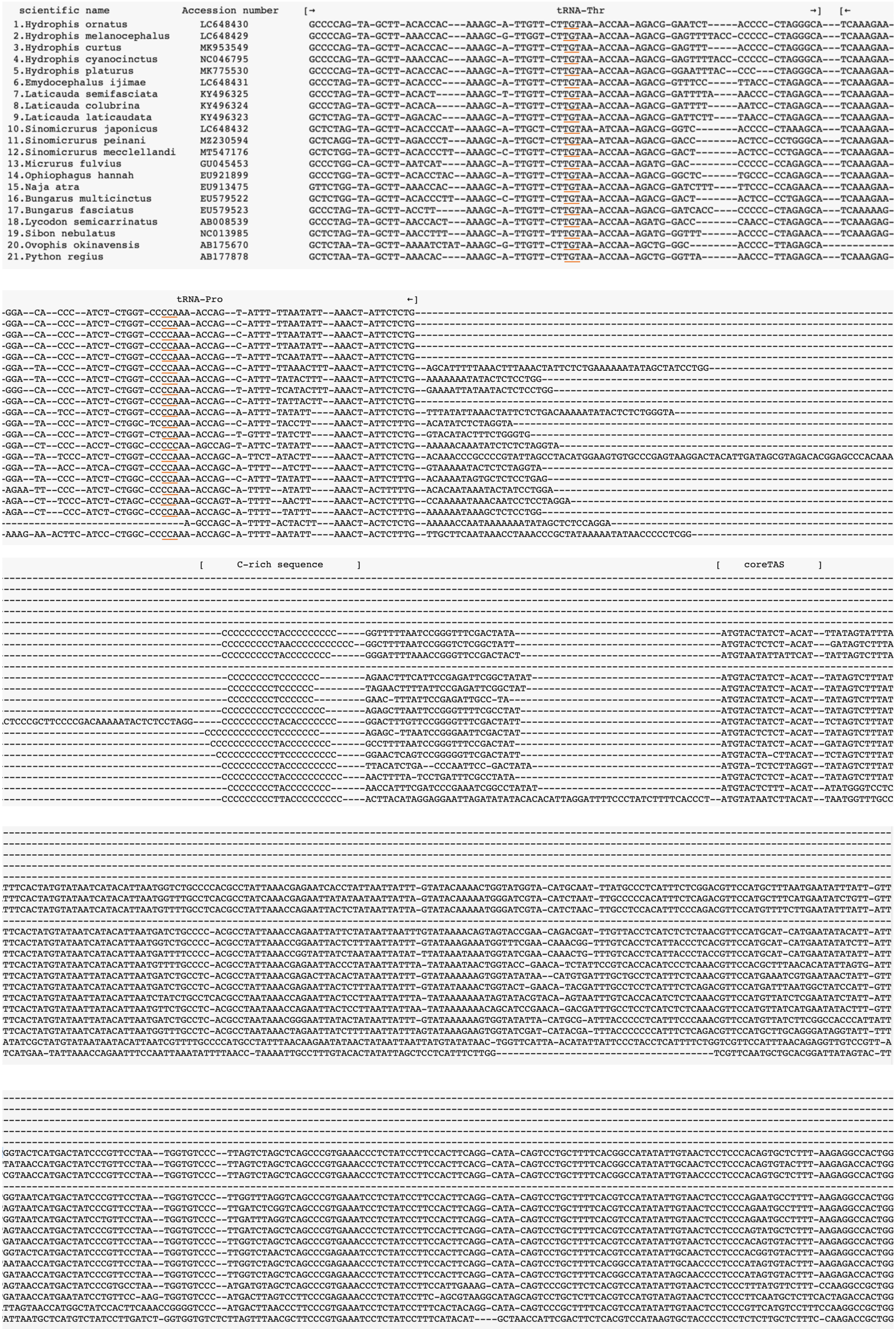

scientific name
Accession number
tRNA-Thr
1.Hydrophis ornatus LC648430
2.Hydrophis melanocephalus LC648429
3.Hydrophis curtus MK953549
4.Hydrophis cyanocinctus NC046795
5.Hydrophis platurus MK775530
6.Emydocephalus ijimae LC648431
7.Laticauda semifasciata KY496325
8.Laticauda colubrina KY496324
9.Laticauda laticaudata KY496323
10.Sinomicrurus japonicus LC648432
11.Sinomicrurus peinani MZ230594
12.Sinomicrurus mecclellandi MT547176
13.Micrurus fulvius GU045453
14.Ophiophagus hannah EU921899
15.Naja atra EU913475
16.Bungarus multicinctus EU579522
17.Bungarus fasciatus EU579523
18.Lycodon semicarrinatus AB008539
19.Sibon nebulatus NC013985
20.Ovophis okinavensis AB175670
21.Python regius AB177878
tRNA-Pro
C-rich sequence
coreTAS

```
------------------------------------------------------------------------------------------------------------------------------------------------
------------------------------------------------------------------------------------------------------------------------------------------------
------------------------------------------------------------------------------------------------------------------------------------------------
------------------------------------------------------------------------------------------------------------------------------------------------
------------------------------------------------------------------------------------------------------------------------------------------------
TTACACTCTCAAGTCCATCTCAACGGCCCGGAACCATCCCTCCCTACTAGCCTTTTCCAACGCTC--TGGTATCACCCGTTATATTGGTACATATCACCTCATGTTCTTATCAGCC-TGCTCGATCCACCCCTGGTAGTCTTTTTT-CTCTCTAC
TTACACTCTCACGTCCATCTCAACGGCCCGGAACCATCCCTCCCTACTAGCTTTTTCCAA-GGCCTTTGGTCGCACCCGTTATATTGGTACATATCACCTCATGTTCTTATCAGCC-TGCTCGCTCCACCCCTGGTAGTCTTTTTTTCTCTCTAC
TTACACTCTCACGTCCATCTCAACGGCCCGGAACCATCCCTCCCTACTAGCTTTTTCCAA-GGCCTTTGGTCGCACCCGTTATATTGGTACATATCACCTCATGTTCTTATCAGCC-TGCTCGTTCCACCCCTGGTAGTCCTTTTT-ATCTCTAC
------------------------------------------------------------------------------------------------------------------------------------------------
TTACACCTTCAAGTCCATCTCAATGGCCCGGAACCATCCCTCCCTACTAGCTTTTTCCAA-GGCCTT-GGTCGCACCCGTTATATTGGTACATATCACCTCATGTTCTTATCAGCC-TGCCAGATCCACCCCTGGTAGTCTTTTAT-CTCTCTCC
TTACACCTTCAAGTCCATCTCAATGGCCCGGAACCATCCCTCCCTACTAGCTTTTTCCAA-GGCCTTTGGTCGCACCCGTTATATTGGTACATATCACCTCATGTTCTTATCAGCC-TGCCAGATCCACCCCTGGTAGTCTTTTTT-ATCTCTAC
TTACACCTTCAAGTCCATCTCAATGGCCCGGAACCATCCCTCCCTACTAGCTTTTTCCAA-GGCCTTTGGTCGCACCCGTTATATTGGTACATATCACCTCATGTTCTTATCAGCC-TGCCAGATCCACCCCTGGTAATCTTTTTT-ATCTCTAC
TTACACTCTCACGTTCATCTCAACGGCCCGGAACCATCCCTCCCTACTAGCTTTTTCCAA-GGCCTTTGGTCGCACCCGTTATATTGGTACATATCACCTCATGTTCTTATCAGCC-TGCCAGATCCACCCCTGGTAGTCTTTTAT-TTCTCTCC
TTACACCTTCAAGTTCATCTCGACGGCCCGGAACCATCCCTCCCTACTAGCTTTTTCCAA-GGCCTTTGGTCGCACCCGTTATATTGGTACATATCACCTCATGTTCTTATCAGCTATGCTAGATCCACCCCTGGTAGTCTTTTAT-CTCTCTCC
TTACACTCTCAAGAACCCCTCAACGGCCCGGAACCATCCCTCCCTACTAGCTTTTTCCAA-GGCCTTTGGTCGCACCCTTTATATTGGTACATATCACCTCATGTTCTTATCAGCTATGCTAGAGCCACCCCTGGTAGCCTTTTTATCTCTCTCA
TTACACTCTCACGTCCATCTCAACGGCCCGGAACCATCCCGCCCTACTAGCTTTTTCCAA-GGCCTTTGGTCGCACCCGTTATATTGGTACATATCACCTCATGTTCTTATCAGCTATGCTAGATCCACCCCTGGTAGTCTTTTTT-CTCTCTCC
TTACACTCTCACGTCCATCTCAACGGCCCGGAACCATCCCGCCCTACTAGCTTTTTCCAA-GGCCTTTGGTCGCACCCGTTATATTGGTACATATCACCTCATGTTCTTATCAGCTATGCCAGATCCACCCCTGGTAGTTTTTTAT-CTCTCTCC
TTACACTCTCAAGAGCATCTCAATGGTCCGGAACCACCCCGCCTTACTTGCTTTTTCCAA-GGCCTTTGGTCGCACCCTTTATAGTGGTACATATCACCTCATGTTCTTATCACCTATGCCAGTTCCACCCCTGGTTGTTCTTTTT-ATCTCTAC
TTACACTTTCAAGGTCATCTCAACGGCCCGGAACCATCCCGCCCTACTTGCTTTTTAAAA-GGCCTTTGGTCGCACCCGTTATATTGGTACATATCACCTCATGTTCTTATCACGTATGCTCGTTCCACCCCTGGTAGCTCTTATT-TAATCTAG
TTACACCTTCAAGATCATCTCAATGGTCCGGAACCACCCCGCCTTACTTGCTCTTTCCAA-GGCCTTTGGTCGCACCCTTTATACTGGTACATTGCACCTCATGTTCTTATCACGTATGCCTGTCCCGCCCCTGGTTGGGGTTTTT---AGGTAC
TTACACTCTCAAGATCATCTCGATGGTCCGGAACCACCCCTCCATACTAGCTTTTTCCAA-GACCTTTGGTCGCACCCTTTATA-TGGTACAATTCACCTCATGTTCTGATCAGCTATGCCAGTCCACCACTGGTATCCCTTTTTT-CTCTCTCC
```

```
                                                                                                                   [         CSB-1            ]
------------------------------------------------------------------------------------------------------------------------------------------------
------------------------------------------------------------------------------------------------------------------------------------------------
------------------------------------------------------------------------------------------------------------------------------------------------
------------------------------------------------------------------------------------------------------------------------------------------------
------------------------------------------------------------------------------------------------------------------------------------------------
CTTTCACCTGACAACCATATATGCCCGTTACC-GTTACCCCT-ACCGGGGTGGACAACCT--AGTCCGGGTGGCGCTTTATTCTTGGTCTTGCATATTCCC-TATATGGATACA--TTCCTTCCATGATTTTAGACATA--TCGTTCTCATTGA
CTTTCACCTGACACCCATATATGCTCGTTACC-GTTACCCCT-ACCGGGGTAGACCATCT--AGTCCGGGTGGGGCTATATTCTTGGTCTAGCATCCTCCC-TATACGGATCA---TTCTTTCCATGTTTGTTAGACATA--TTTTTCCTGGCAC
CTTTCACCTGACACCCATATATGCCCGTTACC-GTTACCCCT-ACCGGGGTGGACAATCT--AGTCCGGGTGGCGCAATATTCTTGGTCTAGCATATTCCC-TATATGGATACA--TTCTCTCCATGATTATTAGACATA--TTTCTCTTATCGC
------------------------------------------------------------------------------------------------------------------------------------------------
CTTTCACCTGACACCCATATATGCCCGTTACC-GTTACCCCT-ACCGGGGTAGACCCTTTT-AGTCCGGGTGGAGCTTGATTCTTGGTCTTACACATTCCC-TATATGGATACA--TCTCTT-CATGGTTCTTAGACATA--TTTTTCTCCTGAC
CTTTCACCTGACACCCATATATGCCCGTTACC-GTTACCCCTCACCGGGGTAGACCCTTT--AGTCAAGGTGGAGCTCGATTCTTGGTCTAGCACATTCCC-TATATGGATATA--TCTCTC-CATGATTTTAGACATA--TTTTTCTCCTCTC
CTTTCACCTGACACCCATATATGCCCGTTACC-GTTACCCCTCACCGGGGCAGACACTTT--AGTCAAGGTGGAGCTCGATTCTTGGTCTTGCACATTCCC-TATATGGATACA--TCCCTC-CATGATTTTAGACATA--TTTTTCTCCTCGC
CTTTCACCTGACACCCATATATGCCCGTTACC-GTTACCCCT-ACCGGGGTAGACCATCT--AGTCCGGGTGGAGCTATATTCTTGGTCTAGCATATTCCC-TATATGGATACA--TTCCTT-CATGCTTGGTAGACATA--CCGTTATCCTATT
CTTTCACCTGACACCCATATATGCCCGTTACC-GTTACCCCT-ACCGGGGTAGACCATCT--AGTCCGGGTGGAGCTTGATTCTTGGCCTAGCACATTCCC-TATATGGATACA--TTCTTC-CATGCTCGTTAGACATA--TTATTTCTCTAAT
CTTTCACCTGACACAGG--TATGCCCGTTATC-GTTACCCCTCGCCGGGGTAGACCCTTT--AGTCAAGGTTGAGCTCGATTCTTGGTCTGGCACATTCCC-TATATGGATACA--TTCCTC-CATGATTATAAGACATA--TTATTCTCTACAT
CTTTCACCTGACACCCATATATGCCCGTTACC-GTTACCCCT-ACCGGGGTAGACCATCC--AGTCCGGGTGGAGCTATATTCTTGGCCTAGCATATTCCC-TATATGGATACA--TTCCTT-CATGCTTGTTAGACATA--TTATTTCTCACAT
CTTTCACCTGACACCCATATATGCCCGTTACC-GTTACCCCT-ACCGGGGTAGACCATCT--AGTCCGGGTGGAGCTTGATTCTTGGCCTAGCATATTCCC-TATATGGATACA--TTCCTT-CATGCTTGTTAGACATA--TTATTCTTCTCGC
CTTTCACCTGACACTCACTT--GCCCGTTACC-GTTCCCC-TCACCGGGGCGTAGACCATCTAGTCCGGGTGGAGCTATATTCTTGGCTATGCACATTCCC-TATACGGATACA--TTCCTT-CATGCTTGTTAGACATA--CTTTTTCCTTGAC
CTTTCACCTGGCACTCACTT--GCCCGTTACC-GTTCCCC-TCACCGGGGCGTCGACACTCTAGTCCGAGTGGAGCTATGTCCATAGCCTGGCTTTCCCCC-TATAGGGTTACA--TTTCCTTAATGCTTGGTAGACATA--TTTTTCCTTTTGT
CTTTCACCTGACACCCATATATGCTCGTTACC-GTCACCCCTCTCCGGGGTAGGTTATT---AGTCCAGGTGGAGCTATACCCTTGGTCGTGCACTTTCTCCTATATGGATACA--TCTC-TTAATGCTTGTTATACATA--TTCTTCACTACTG
CTTTCACCTGACTACCATATATGCACACACACAGTTAGGCATTTAGTCCG--GG-TTG----AGCACTA-TGTATCCCTCCACACCTTCCCTTGAGGCTTA-CA------------TCTC-TTAATGCTTGTTAGACATA--TATCTTTTCCCC
```

```
                                                                                                                [        CSB-3       ]
------------------------------------------------------------------------------------------------------------------------------------------------
------------------------------------------------------------------------------------------------------------------------------------------------
------------------------------------------------------------------------------------------------------------------------------------------------
------------------------------------------------------------------------------------------------------------------------------------------------
------------------------------------------------------------------------------------------------------------------------------------------------
CCACTTTTCT-TCACTATTTCTTTATTACAAAATTGCCGGAGTTCACATTTTTTTTTAAGCCCTATTTTTAAAAATCAT-GTTTCGGCTTG-CGACGACTTTATAAT-------AAAACCCTCTCCTAGAAA---TTCTATAC-TATTATTTC
CGACTTTTCT-CACTATTTTTTATTATACTTTCA-CCGCTGTTGGCACTTTTTTTAAACCCCTATTTTTAAAAATCACACCTTCAAGATA-CGACAATATACAATA-------AAAACCCTCCCCCAAAAA---AGTATAGTTATTACTTTCA
CGGCTTTTCT-CATTGTTTTTTATAGCATTTTTA-CCGCTGTTTGCACAATTTTTAAACCCT-ATTTTTAAAAATCTTACTTTCACGATA-CGACAAAACTATAAT-------AAACAACGTTCCCAAAAA---AGTATAATTATTTTTATAC
------------------------------------------------------------------------------------------------------------------------------------------------
TCACTTTTCT-CATTATTTTATTATAGTAATTTCG-CCGCTGTTAGCACTTTTTTTAAAGGCTCATTTTTAAAAATCATACCTTTTTAATA-CGACAAACTA-CAAT-------AAAATTGCCTTCCCAAAA---TAATATAATTATACTTACA
CCGCTTTTAT-CATTATTTTTTTTAGTATTTTCG-CCGCTGTTAGCACTTTTTTTAAACCCCTATTTTTAAAAATCACACTTATGCAATA-CGATAAACCCCAATAAA-----AAATACCCCCAAAATAGT--ATAATTATATTTTCATATGA
CTACTTTTTC-CATTGTTTTTTTTTATAGTTTCG-CCGTTGTTAGCACTTTTTTTAAAACCCCATTTTTAAAAATCACACCTACGCAATA-CGATAAACTACAGTAAA-----ATATACCCCCAAAATAAT--ATAATTATTATTTCATACGA
CCGCCTTTTTACATTATTTTTTTAATGGTTTTTTC-CGCTGTTAGCTAATTTT-CAAAACCCCTATTTTTAAAAATCAAGCCTCTGATATA-CGACAAAAC-ATAAT-------AAAAACAATGTCCCAAAA---TAGTACACTTTCTCACACA
CCACTTTTTC-CATTATTTTTTTATAGTAATTTCA-CCGCTGTTAGCACTTTTTTTAAACCCCTATTTTTAAAAATCACACTATTGCAATA-GGTCAAAACTATAAT-------AAAAACACATAACCAAAA---TAATATAATTATTTTTTCA
CCACTTTTTC-CATTATTTTTTTATAGTAATTTCA-CCGCTGTTAGCACTTTTTTTAAACCCCTATTTTTAAAAATCATGCCTCTCTAATA-CGATAAAACTATAAT-------AAAATCATAATCCCAAAA---AAATATAATTATTTT-CAA
CCACTTTTTC-CATTATTTTTTTATAGTTATTTCA-CCGTTGTTCACACTTTTTTTAAACCCCTATTTTTAAAAATCATGCTTCTACAATA-CGACAAACCTATAATAA-----AACTAACCCCATAAATAT----TACAATCTTTTTTACATC
CGACTTTTTT-CATTGTTTTTTTATAGTAATTTTA-CCGTTGTTCACACTTTTTTTAAACCCCTATTTTTAAAAATCATGCTTCTACAATA-CGACAAAACTATAAT-------AAAGCCACTACCCAAAAA---AAGTATAATTTTTTTTACA
TGTAAAATTT-CATTATTCTTTTATTATAAATTTG-CCGCAGTAAACACTTTTTCATCACCCCTATTTTTAAAATCTGAGAAAAAATAATA-CGACAAAACTATAAT-------AAACCCCCCGCCCCGAAA----CCCCATATACTTATTTC
CGCAGCTCACTCATTATTTTTTTATTGTCAATCTG-CCGCTGCTTGCATATCTTCTGCT-CCTCATTTTTCAGAAATATGACAATTTTAATACGACAAAGTTATAAT-------AAATAATAATGTCCCAAA----ATTATATACTGGTGTATA
CTGAAAATTT-CATTATTTTTTACTAAAGAAATCCCGGTGTAAATACACTTTTT---CACCC--ATTTTTTCAAATTTTACCAAAATCAA-TACCACTTTTCTATACT-----AAAATTTCAAACCCGAAA----TCCTATAAGAACTTATTT
CCGAAATTTTACACACCAAAACTTCG-------------------------------------------------------------------------------------AAAAAAACCCCCAACTTT----TAAGAACTTCCTACTCAC
```

```
------------------------------------------------------------------------------------------------------------------------------------------------
------------------------------------------------------------------------------------------------------------------------------------------------
------------------------------------------------------------------------------------------------------------------------------------------------
------------------------------------------------------------------------------------------------------------------------------------------------
------------------------------------------------------------------------------------------------------------------------------------------------
ATGTTATCTTTGAATACCCCTCACATCACACTTACCTAATTTTAACCCCGGGTTTTCACTACTTTTAATCCTAAAAGTCTATTAAAAAAATAAAAACTTTCCACTACGCTACTATAGCATTTTTATCTTTAA----------------------
TATAACCCACACGCCCGTTTATATATT---------------------------------------------------------------------------------------------------------------------
AACCTTACATTCAACCCCCCCCATACTCCTACTATTTTAATGGTTTTTCCGTATCTATATTACAATACTGATTTTTTATAATAAAA------------------------------------------------------
------------------------------------------------------------------------------------------------------------------------------------------------
TAAGAACTGTTTTGCCACGACACTTTATCTTATTATATAATTTTCCCATGCGAATTTGTTAATCGAAATTTTGCCACTTTCTCTTCAAATTAAATTCTAATGTTAAAATACTTCCTCTCGTAATTAAAGACTTTTTCCCCATATTACAACTTAAT
TCTGTTATTACCGATATCTCATCTTATTATATAATTTTCCCACATGAATTTTCTTTCCAGAGATTTGCCACTTTTTCTCCAAAAGTTTAATTCAGATGTGAAAAAACTCTCCTCAAAATTAAAGTCTTTTCTCCCATAATTACAGTTTAATACGA
TCTCTTTTTACAGATACCTCACCTTATTATATAATTTTCCCATGTAAATTTTCTTTCCGGAATTTTGCCACTTTTTCTCCAAAATTAAATTCAAATGTATAAAAAACCTCCCCCAGAATTAAAGGACTTTTCCCCTTTAATCACGATTTAATCC
TATCATCCTCACCACAGTATGATTATACTTTTTTCCCATCTTGAAATTTACCCCGGAAATCTGCCATTATTTCCTTGGATTAAACTTCAATTATCAGAATTACCCYGTGACAAATGTGGTAATTCCCCCTTGTTTCTTAATACTGTGCGAGAATT
TACAACCTCTCACACCCCCTGACACCTCCATTATTAAATAATTTTCCCACACAAACTTTTAATCCGAAATTTTGCCACCTTTTTCAGAAATTAAAATTCAAATGTCAAAATCACCCCCTCACCGTTATAGCAATTTCTCCCGCAAAGGGGTTATA
TTTCTTTTACATATTACCTCTCACCTCTCCCGACACCCCATTTTATTAAAAATTTTCCCAAGCTAATTCTCATTCCAGAAATTTGCCACCTTTTTCCAAAACAAAAATTCAAATGCCCAAATCCCCCCGACACCCTTATAGTCACTTTTCCCTT
CTTCTCTCTCTCTCTCTCACCCT-CTTTTTAATATAAAATTTTCCCATGCTTATTTTTAATCCGAGTATTCACTATCTTATCTCCAATTTTAAATTCAAATGTCAAATTCACCCCTTCACCAATATAGTTTTTTTTTCCTTTTTACCTGTCACAA
TTCCATCTTTATTATCACTCACTCTCTTTTTATTAAAAAACTTTCCCATGTTTAATTTTAACCCAGAAATTTGCCACTTTTCATTTAAATTTTAATTCAAGTGTCAAATTAACCCCTTCACCATTATAGTCATCTTCTCTTTAATTACATTACAA
ATACCAGCCTCTCACCCCGATCACACCCCGTGAAAATAGTTTACTATAAAAATAGCAAACACATATTTTAGAACCGCATATTTAACTTCTTTCTCTGAAAAATCATTTCAAATGCCAAAACACCCCCCACTAAAAAATAGTCATTTCACCAATTA
CAAATACCTCTCACCGCCAATTTCACGCCCGTAAAAAAAAGTTGCTATAAAAATCACAAACATAAATTTAGTGTCCTATATTTCGCCATCTTTCCTGGGGAGAGAATTTCAAACGTTGAAATCGGCCCCCCCAAAAAAATAGTCATTTTACCAAC
TTCATTTCACCGTTTTATTTTTTTTACACGAGAATAAGAATATTATAAAAAACACGGTTCAACTTTTAAGTAGCCGAAAATTCACTATTTCTCCAAAATTTCAATTCATAAGTGCAAATCACCCCCAAAAAATTACAGCCGATTTTGCCTTTATT
CACCAAAATAAAACTAAACCTATAATTACGACCCGCCGTTCACTCCTACACTCACATCTCTTTACCTTAAAACAATACAAAACGGGCCTAATCACAAGCCCCGCCTTCATGAAACTTTTACTTCTATGATTACTCACCACAAAATAAAAATATAC
```

```
------------------------------------------------------------------------------------------------------------------------------------------------
------------------------------------------------------------------------------------------------------------------------------------------------
------------------------------------------------------------------------------------------------------------------------------------------------
------------------------------------------------------------------------------------------------------------------------------------------------
------------------------------------------------------------------------------------------------------------------------------------------------
------------------------------------------------------------------------------------------------------------------------------------------------
------------------------------------------------------------------------------------------------------------------------------------------------
------------------------------------------------------------------------------------------------------------------------------------------------
------------------------------------------------------------------------------------------------------------------------------------------------
ATGCTGATTTTATATAGTAATAA-------------------------------------------------------------------------------------------------------------------------
AGATTTATATACAAAT--------------------------------------------------------------------------------------------------------------------------------
GAAATTCATATAATAAAAAT----------------------------------------------------------------------------------------------------------------------------
TTTATAATAATAAATATTATTTTCACCTACAAATTAAACGAAAAATTTCCTATAAAAATTATCTCTTGAGATTGATTTTTACATTTTAAATTTTTTTTTCTCCAAAAACGTGGCCATTTCCCCTTGCTTTCAACACTATGCGGAGATTAATAATA
AATGGGAGAATTATATATAAAA--------------------------------------------------------------------------------------------------------------------------
TATTGGCATCATAATACGGGATTTGTATACTGAATCTACAACAAAAAT------------------------------------------------------------------------------------------------
ATGCTAAATTTCTATACAATTATAACTTTCAACTTATATTTTAGCCCATAAAAGTACT--------------------------------------------------------------------------------------
ATACTAAATTTTTATACAATTTTTTTAAAAGCTTACTGTTAAAGGCATCAAAATATTAAATTCAATACTATATAATCCTCAACAAAACCCTTAATTTATTAAACATAA-------------------------------------
ATCTCAACAATCTCCCGAATTTTTATATAA------------------------------------------------------------------------------------------------------------------
CTAAAATCGTATAGTCCTAAATTTTTATATTAA---------------------------------------------------------------------------------------------------------------
TTTCAACCCAAACCCGAATTTCTATATATTTGC---------------------------------------------------------------------------------------------------------------
GGAAAACAAACCTCTAATCACGATTTGACGTTTGTTCTTATATAATTACTTCTATTCTACACACTACCAAAGCAAATCCAACCATCATCCACTTTCTCTACACCACCCAAAA---------------------------------
```

```
                                                           [→repeats region
--------------------------------------------------------------------------------------------------[AAACAAAACTCTCATGCC-ATACTACAAT-----------------ATAGCACCCATTTCA
----------------------------------------------------------[CCCCAAAACCAGCATTTTTAATATTAAACTATTCTCTGAAACAAAGCTTTCATGCC--CACTATAAT-----------------A--GTATTTATTTCA
---------------------------------------------------------------------------------------------------AAGCAAAACTTTCATGCC-CTACTATAAC-GTACTACTCACACTCGATAGTGCCTATTTCA
---------------------------------------------------------------------------------------------------AAACAAAGCTTTCATGCCC--ACTATAATAGTATATAATCATACATTAATAATCATC--CT
----------------------------------------------------------------------------------------------------AATTAAAATCTCCTGCCCC-CACTATAATGTTCTACCCATACTCAATAATATCTATTTCA
-----------------------------------------------------------[TTATAAAATAATCCGAGATTAATATATAAATATATTCTCCCACCAGCCTCCCTCCCCCAAAAAAAAAGGTACAAGCATGTTTCGTCTCGATGTAATTT
-----------------------------------------------------------[TTTTTCCCACGCCCAATTTTATTCCGGGTTTTTGCCACTTTTCCACAGAATTAATTTTCAACTGCTAAAACCCCCCTCAACTACTATAGTGATTTTTA
-------------------------------------------------------------------------------------------------------------------------------------------------------------------
-------------------------------------------------------------------------------------------------------AAAGA---CACGCTCTCCTTGACCCAGCTAATCCAAAAATTAACTATCTGTACTACT
-------------------------------------------------------------------------------------------------------------------------------------------------------------------
-------------------------------------------------------------------------------------------------------------------------------------------------------------------
-------------------------------------------------------------------------------------------------------------------------------------------------------------------
AAAAATATTTTCACGCACATAAAGTACCTAACAACTTCCCTGCCCATTTATACAAA-------------------------------------------------------------------------------------------------------------
-------------------------------------------------------------------------------------------------------------------------------------------------------------------
-------------------------------------------------------------------------------------------------------------------------------------------------------------------
-------------------------------------------------------------------------------------------------------------------------------------------------------------------
-------------------------------------------------------------------------------------------------------------------------------------------------------------------
-------------------------------------------------------------------------------------------------------------------------------------------------------------------
-------------------------------------------------------------------------------------------------------------------------------------------------------------------
-------------------------------------------------------------------------------------------------------------------------------------------------------------------
-------------------------------------------------------------------------------------------------------------------------------------------------------------------
```

```
CTAT--ATAACCACACATTAAG---TGCCCTACTTTCCC--CGAA--ATTTTACTATTGTTTACCACAAAAATTTATTAAATGTTAAAATATCTCTAAGTTCTGCTTTTATCAAATAAACATACTAAATTCATATATAAATATCCTTATTTTTCA
CTAT--ATAATCATACATTAATAATCATCCTACTGTTCCACCGAA--ATTTTACTACAAAAACCCATTAAATGTTAAAATATCCCAAAAATAATACAAGTTCTGTTTTTATTAAATAAACATACTAAATTTATATACAAACATACTTATTTTTC-
CTATGTATAACCATACATTAACA-CCACCCTATTCTTCC--CCAA--ATTTTACTACAA--AATTATTAAATATTAAAATATAACACAA--------AGTTCTGTTTTTATTAAATAAACATACTAAATTTATATATATAAATATACTTTATTATCA
ACT-GTTCCMCSRAAWTTTACTACMWACAT-TTATAGTATTC-----ATTTCACTATGTATAATCATACATTAATGGTCTGCCCCACGCCTATCAAACGAGAGCCTT--ATAATAATTATTTGTATACAAAAGTAGTAT----------------
CTACATAAAATCATACAT-ATAACCACCCTACCCTTTCTCTCCCAAGATTTTAATACTTTTTA-----------------CCACAAAATTATTAAATATTAAAATATCTCTCATATAACACTAAGTTCTGTTTTTATTAAACAAACATACTAAA
TTTATTCCCAGTTTTTACTATTATTAGTTCTAACAAGCCTATTTAAAAATATTAAAATTACTTCTCACACTACTATAGCATTTTTACCTTTA] 8 tandem repeats of 190bp sequence---------------------------
CTTTACCTTGCTGCATACAAGATTTATATACAAAAAGTTATTATAGCTTATCACTAAGCACAGCACTAAAATGCTAAAAATGACCCGCTACTAACAACACTAGGTCTTAGTTTTAACCACACCAAAACAGATGCAGCTACAGCCATTTATATGTT
-------------------------------------------------------------------------------------------------------------------------------------------------------------------
A-ACATTAGCTAGTTCTATTAACA-----TTATAGTCTTTATTTCACTATGTA-TAATCATACATTAA----TGATAT---GCCTCACGCCTATTAAACCGGTATTATTCCAATAATTATTATTATACAAAAATGGTATTGTACATCTAACTTGT
-------------------------------------------------------------------------------------------------------------------------------------------------------------------
-------------------------------------------------------------------------------------------------------------------------------------------------------------------
-------------------------------------------------------------------------------------------------------------------------------------------------------------------
-------------------------------------------------------------------------------------------------------------------------------------------------------------------
-------------------------------------------------------------------------------------------------------------------------------------------------------------------
-------------------------------------------------------------------------------------------------------------------------------------------------------------------
-------------------------------------------------------------------------------------------------------------------------------------------------------------------
-------------------------------------------------------------------------------------------------------------------------------------------------------------------
-------------------------------------------------------------------------------------------------------------------------------------------------------------------
-------------------------------------------------------------------------------------------------------------------------------------------------------------------
-------------------------------------------------------------------------------------------------------------------------------------------------------------------
-------------------------------------------------------------------------------------------------------------------------------------------------------------------
```

```
CGTTTTTCCCCGAAAAACCAC---------ACAAACATATTTTACTCCCAATTTTTACTATTGTGTATTTTAAAAACTTAAAAGTGTTAGATTTACTCTCACGCTACTATGGTAATTTTATCCTTCTTAATAAAATAATATTAAACTATT-TATA
--------CCTGAAAAATAAC---------ACACACATATTTTACTCCGGTTTTTATTATTACCTGTTATAAAAACT-AAAAAAATTAAATTTACTCTCACACTACTATAGCAATTTTATCTTTATTAATA-------------------TACA
CCATTTTTTCAAAGGAGTCCAT--GCTTTAATGAATATCTGTTGTTGGTACCCATGACTATCCCGTTCCTAATGGTGTCCCTTAGCCTAGCTCAGCCCGTGAAACCCTCTATCCTTCCACTTCAGGCATACAGTCCTGCTTTTCACGGCCATATA
-GA----TACATAMCGTTCCAC--GCTTTAATGGATATCTATTGTTGATACTCATGACTATCCCGTTCCTAATGGTGTCCCTTAGCCTAGCTCAGCCCGTGAAACCCTCTATCCTTCCACTACAGGCATACAGTCCTGCTTTTCACGGCCATATA
TTTATATACTAATATATTAATT--------------------ATCACCATTTTTTTACTATTTCCCCG-AAAAACAACA--------------------CAAACATACTTTAATCCCATTTTTATTATTAAGTAC--CCTAAAACTTAAGAACA
-------------------------------------------------------------------------------------------------------------------------------------------------------------------
] 4 tandem repeats of 253bp sequence----------------------------------------------------------------------------------------------------------------------------------
-------------------------------------------------------------------------------------------------------------------------------------------------------------------
CTCCACATTTCTCAAACGTTCCATGCTTTATTGCATATTTGTTATTTATAATCATGACTATCCCGTTCCTAATGGTGTCCCTTGATCTAGCCAAGCCCGTGAAACCCTCTATCCTTCCACTTCAGGCATACAGTCCTGCTTTTCACGGCCATATA
-------------------------------------------------------------------------------------------------------------------------------------------------------------------
-------------------------------------------------------------------------------------------------------------------------------------------------------------------
-------------------------------------------------------------------------------------------------------------------------------------------------------------------
-------------------------------------------------------------------------------------------------------------------------------------------------------------------
-------------------------------------------------------------------------------------------------------------------------------------------------------------------
-------------------------------------------------------------------------------------------------------------------------------------------------------------------
-------------------------------------------------------------------------------------------------------------------------------------------------------------------
-------------------------------------------------------------------------------------------------------------------------------------------------------------------
-------------------------------------------------------------------------------------------------------------------------------------------------------------------
-------------------------------------------------------------------------------------------------------------------------------------------------------------------
-------------------------------------------------------------------------------------------------------------------------------------------------------------------
-------------------------------------------------------------------------------------------------------------------------------------------------------------------
```

```
ACACAAATGTTAACACAGCTTACA-----GCACAGCACTGAG-ATATGCTAAA--CAACCTTACCTGTTAACAACCAATCGT----GCAGCGTGTTATCACAGCCCTTATTAAGAGCA--GCACTGAAAA-TGCTAAATCACCCTAACTTTA-TA
ACACAAATGTTAACACAGCTTGCAACCAAGCACAGCACTGAG-ATGTGCTAAAA-CGACCTTACCTGTTAACAACCAATCGT----ATAAAATGTTATCATAGCCCTTGTTGGGA-CA--GCACTGAAAAATGCTAAATTACCCCAACTTCA-TA
TTGTAACTCCTCCCACAG--TGCTCTTTAAGAGGCCACTGGTTACACTCTCACGTCCATCTCAACGGCCCGGAACCATCCCTCCCTACTAGCTTTTTCCAAGGCCTTTGGTCCACCC-GTTATATTGGTACATATCACCTCATGTTCTTATCAGC
TTGTAACCCCTCCCACAG--TGCTCTTTAAGAGGCCACTGGTTACACTCTCACGTCCATCTCAACGGCCCGGAACCATCCCTCCCTACTAGCTTTTTCCAAGGCCTTTGGTCGCACCCGTTATATTGGTACATATCACCTCATGTTCTTATCAGC
TTTAAATTACTCTCACACTACTGT----------AATGATTCTATTCTTAT---TAATAAAATAGTGTTAAATTAATAAACAC---AAATGTTAACACAGCTTACAACC-AAGCACAGCACTTAGACATGCTAAA-ACGACCTTACCTGTTAA-C
-------------------------------------------------------------------------------------------------------------------------------------------------------------------
-------------------------------------------------------------------------------------------------------------------------------------------------------------------
-------------------------------------------------------------------------------------------------------------------------------------------------------------------
TTGTAACTCCTCCCACAG--TGTACTTTAAGAGGCCACTGGTTACTCTTTCAGGTCCATCTCAACGGCCCGGAACCATCCCTCCCTACTTGCCTTTTAAGAGGCCTTTGGTCTCACCCGTTATATTGGTACATATCACCTCATGTTCTTATCAGC
-------------------------------------------------------------------------------------------------------------------------------------------------------------------
-------------------------------------------------------------------------------------------------------------------------------------------------------------------
-------------------------------------------------------------------------------------------------------------------------------------------------------------------
-------------------------------------------------------------------------------------------------------------------------------------------------------------------
-------------------------------------------------------------------------------------------------------------------------------------------------------------------
-------------------------------------------------------------------------------------------------------------------------------------------------------------------
-------------------------------------------------------------------------------------------------------------------------------------------------------------------
-------------------------------------------------------------------------------------------------------------------------------------------------------------------
-------------------------------------------------------------------------------------------------------------------------------------------------------------------
-------------------------------------------------------------------------------------------------------------------------------------------------------------------
-------------------------------------------------------------------------------------------------------------------------------------------------------------------
-------------------------------------------------------------------------------------------------------------------------------------------------------------------
```

```
ACA-TAGGTCTTAGTTTTAAACCTCTTATTATCTAAACTCTCTACTT---ACACATGCAAGCCTCAACACAACGAGATAGCCCACTACAACCACCCAAAGGAGCCG-TATCAAATAGTATGCCCCGAACACCATCTTTGATCCCCCAAACCAGTA
ACACTAGGTCTCAGTCTTAAACCTCTTATCATCTAAACCCTCTACTT---ATACATGTAAACCTCAACACA----------------CAACCGCCTAAGCAGACTGGTATCAGAAAAAGC] 5 tandem repeats of 592bp sequenc
TATGCTCGTTCCACCCCTGGTAGTCTTTTTTCTCTGTACCTTTCACCTGACACCCATATATGCCCGTTACCGTTACCCCTACCGGGGTAGACCATCTAGTCCGGGTGGCGCTTTATTCTTGGTCTAGCATATTCCCTATATGATTATATTCTTTC
TATGCTCGTTCCACCCCTGGTAGTCTTTTTTCTCTCTACCTTTCACCTGACACCCATATATGCCCGTTACCGTTACCCCTACCGGGGTAGACCATCTAGTCCGGGTGGCGCTTGATTCTTGGTCTAGCATATTCCCTATATGATTATATTCTTTC
AACCAAACTTGTAGAATGTTATTATAGCCCCCCATTAAGGGCAGCACTAAAAATGCTAAATA-----ACCCC----------AACTTTATAACACAGGG----------TCTTAGTTT--TAAACCTCTTAT-TATCTAAACTCTCTAC------
-------------------------------------------------------------------------------------------------------------------------------------------------------------------
-------------------------------------------------------------------------------------------------------------------------------------------------------------------
-------------------------------------------------------------------------------------------------------------------------------------------------------------------
C-TGCTCGCTCCACCCCTGGTAGTCTTTTT-CTCTCTACCTTTCACCTGACACCCATATATGCCCGTTACCGTTACCCCTCACCGGGGTAGACACTTTAGTCCGGGTGGCGCTAGATTCTTGGTCTGGCATAATCCCCCATATGGATACATTCCT
-------------------------------------------------------------------------------------------------------------------------------------------------------------------
-------------------------------------------------------------------------------------------------------------------------------------------------------------------
-------------------------------------------------------------------------------------------------------------------------------------------------------------------
-------------------------------------------------------------------------------------------------------------------------------------------------------------------
-------------------------------------------------------------------------------------------------------------------------------------------------------------------
-------------------------------------------------------------------------------------------------------------------------------------------------------------------
-------------------------------------------------------------------------------------------------------------------------------------------------------------------
-------------------------------------------------------------------------------------------------------------------------------------------------------------------
-------------------------------------------------------------------------------------------------------------------------------------------------------------------
-------------------------------------------------------------------------------------------------------------------------------------------------------------------
-------------------------------------------------------------------------------------------------------------------------------------------------------------------
-------------------------------------------------------------------------------------------------------------------------------------------------------------------
```

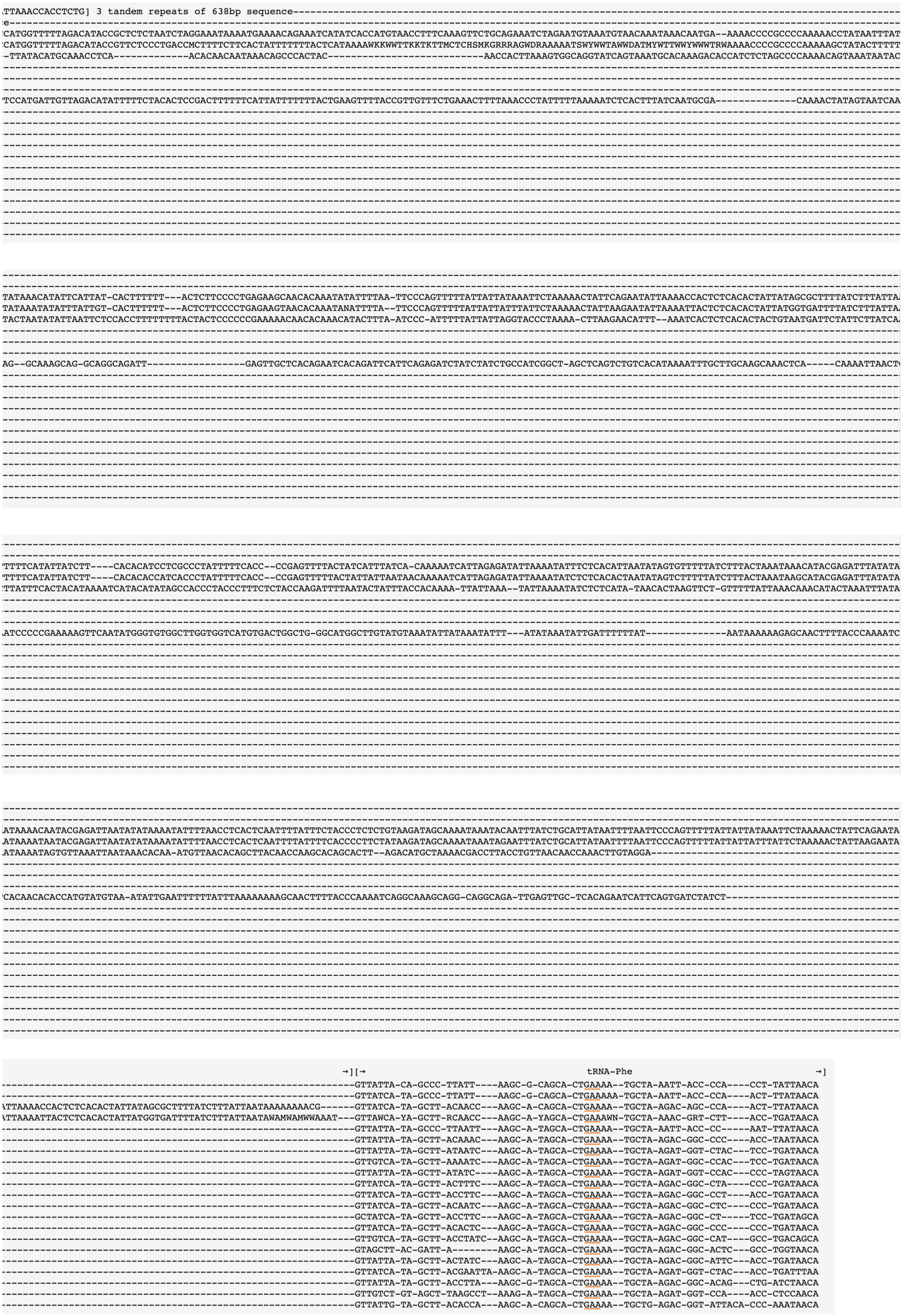

Supplementary figure S3. Sequence comparison of the MNCR2 region among 21 snake species. The control region sequences were aligned with the aid of ClustalX software after identifying common sequence blocks (C-rich, CSB-1, CSB-3, and coreTAS sequences). Transfer RNA genes were aligned based on their clover-leaf secondary structures. The red line indicates the anticodon of each tRNA gene. Sequences for tandemly duplicated regions are represented by a single repetitive unit sequence with a note on repetitive numbers.

scientific name	Accession number	[→ tRNA-Ile →]
1.Emydocephalus ijimae	LC648431	GGAAGCG-TG-CCCG-AGC-----AAGG-A-CTACA-TTGATAG-AGTAG-ACAC--GGAG--ACCATAAA------CTCC-CACTTCCC
2.Hydrophis ornatus	LC648430	GGAAGCG-TG-CCCG-AGT-----AGGG-G-CTACA-TTGATAG-AGTAG-CCAC--GGAG--ACCGAAAA------CTCC-CACTTCCC
3.Hydrophis melanocephalus(1)	LC648429	GGAAGTG-TG-CCCG-AGT-----AGGG-A-CTACA-TTGATAG-AGTAG-ACAC--GGAG--ACCGAAAA------CTCC-CACTTCCC
4.Hydrophis melanocephalus(2)	LC648429	AGAAGTG-TG-CCCG-AGT-----AGGG-A-CTACA-TTGATAG-AGTAG-ACAC--GGAG--ACCGAAAA------CTCC-CACTTCCT
5.Hydrophis curtus	MK953549	GGAAGTG-TG-CCCG-AGT-----AAGG-G-CTACA-TTGATAG-AGTAG-ACAC--GGAG--ACCGAAAA------CTCC-CACTTCCC
6.Hydrophis platurus	MK775530	GGAAGCG-TG-CCCG-AGT-----AGGG-A-CTACA-TTGATAG-AGTAG-CCAC--GGAG--ACCGAAAA------CTCC-CACTTCCC
7.Hydrophis cyanocinctus	NC046795	GGAAGTG-TG-CCCG-AGT-----AGGG-A-CTACA-TTGATAG-AGTAG-ACAC--GGAG--ACCGAAAA------CTCC-CACTTCCC
8.Laticauda semifasciata	KY496325	AGAAGCG-TG-CCCG-AATTA---AAGG-A-CTACC-TTGATAG-CGTAG-ACAT--GGAC--CTCCCTCA------ACCA-CAACCACA
9.Laticauda colubrina	KY496324	GGAAACG-TG-CCCG-AGT-----AGGG-A-CTACC-TTGATAG-AGTAG-ACAC--GGAG--ACCAACAAA-----CTCC-CGCTTCCT
10.Laticauda laticaudata	KY496323	GGAAGCG-TG-CCCG-AGCA----AAGG-A-CTACA-CTGATAA-AGTAG-ACAC--GGAG--CCTATAAA------CTCC-CGCCTCCC
11.Sinomicrurus japonicus	LC648432	GGAGGCG-TG-CCCG-AGCA----CAGG-G-CTACC-TTGATAG-AGTAG-ATAC--GGAA--CCCATAAA------TTCC-CACCTCCC
12.Sinomicrurus peinani	MZ230594	GAAGACG-TG-CCTG-AGT-----AAGG-A-CTACC-TTGATAG-AGTAG-ACAC--GGAA--CCCAGAAA------TTCC-CGTCTTCC
13.Sinomicrurus mecclellandi	MT547176	GGAGGTG-TG-CCTG-AGA-----AAGG-A-CTACA-TTGATAA-AGTAG-ACAC--GGAG--CCATACCTCCCAC-CTCC-CACTTTCC
14.Micrurus fulvius	GU045453	GGAAGCG-TG-CCCG-AGAA----AAGG-A-CTACC-TTGATAG-CGTAG-ACAC--GGAG--CCAATAAA------CTCC-CGCTTCCC
16.Ophiophagus hannah	EU921899	GGAAGTG-TG-CCCG-AGT-----AAGG-A-CTACA-TTGATAG-CGTAG-ACAC--GGAG--CCCACAAA------CTCC-CGCTTCCC
15.Naja atra	EU913475	GGAAGCG-TG-CCTG-AGT-----AAGG-A-CTACC-TTGATAG-TGTAG-ACAC--GGAA--CCCACAAA------TTCC-CGCTTCCC
18.Bungarus multicinctus	EU579522	GGAAGTG-TG-CCTG-AGT-----AAGG-A-CTACT-TTGATAG-CGTAG-ACAC--GGAA--CCCAATAAA-----TTCC-CACCTCCT
17.Bungarus fasciatus	EU579523	GGAAGTG-TG-CCCG-AGCT----AAGG-A-CTACC-TTGATAG-AGTAG-ACAC--GGAA--CCAATAAA------TTCC-CACTTCCC
19.Lycodon semicarrinatus	AB008539	GGGGGTG-TG-CCCG-AGT-----AGGG-A-CTACC-TTGATAG-AGTAG-ACAC--GGAA--CCATAAAA------CTCC-CACCCCTC
20.Sibon nebulatus	NC013985	GGAAATG-TG-CCCG-AGTC----AGGG-A-CTACC-TTGATAG-AGTAG-ATAC--GGAA--CTAAGCA-------CTCC-CACTTCCC
21.Ovophis okinavensis	AB175670	GGAAGTG-TG-CCTG-AGAACAC-AAGG-A-CTACC-TTGATAG-AGTAG-ACAC--GGGA--ACCACA--------ACCC-CACCTCCC
22.Python regius	AB177878	GGAAGTG-TG-CCCG-AGAC----AGGG-A-TTACC-TTGATAG-AGTAA-ACAC--AGGA--TTACAA--------CCCT-CACTTCCT

Supplementary figure S4. Sequence comparison of tRNA^{Ile} genes among 21 snake species based on their clover-leaf secondary structures. *Hrdrophis melanocephalus* mtDNA encodes two copies of tRNA^{Ile} genes (noted with 1 and 2) that correspond to I_1 and I_2 in Fig. 3, respectively. The red line indicates the anticodon.

scientific name	Accession number	[→ tRNA-Phe →]
1.Hydrophis melanocephalus(1)	LC648429	GTTATC-A-TA-GCTT-ACAACC---AAGC-A-TAGCA-CTGAAAA--TGCTA-AGAC-GGC-CTT---ACC--TGATAACA
2.Hydrophis melanocephalus(2)	LC648429	GTTATC-A-TA-GCCC-TTATT----AAGC-G-CAGCA-CTGAAAAA-TGCTA-AATT-ACC-CCA---ACT--TTATAACA
3.Hydrophis melanocephalus(3)	LC648429	GTTATC-A-TA-GCCC-TTGTT-----GGG-A-CAGCA-CTGAAAAA-TGCTA-AATT-ACC-CCA---ACT--TCATAACA
4.Hydrophis melanocephalus(4)	LC648429	GTTATC-A-TA-GCCC-TTATT----AAGC-G-CAGCA-CTGAAAAA-TGCTA-AATT-ACC-CCA---ACT--TCATAACA
5.Hydrophis melanocephalus(5)	LC648429	GTTATC-A-TA-GCCC-TTGTT----GGGC-G-CAGCA-CTGAAAAA-TGCTA-AATT-ACC-CCA---ACT--TCATAACA
6.Hydrophis melanocephalus(6)	LC648429	GTTATC-A-TA-GCCC-TTGT-----TGGG-A-CAGCA-CTGAAAAA-TGCTA-AATT-ACC-CCA---ACT--TCATAACA
7.Hydrophis ornatus(1)	LC648430	GTTATT-A-CA-GCCC-TTATT----AAGC-G-CAGCA-CTGAAAA--TGCTA-AATT-ACC-CCA---CCT--TATTAACA
8.Hydrophis ornatus(2)	LC648430	GTTATC-A-CA-GCCC-TTATT----AAGA-G-CAGCA-CTGAAAA--TGCTA-AATC-ACC-CTA---ACT--TTATAACA
9.Hydrophis ornatus(3)	LC648430	GTTATT-A-CA-GCCC-TTATT----AAGG-G-CAGCA-CTGAAAA--TGCTA-AATC-ACC-CTA---ACT--TTATAACA
10.Hydrophis cyanocinctus	NC046795	GTTAWC-A-YA-GCTT-RCAACC---AAGC-A-YAGCA-CTGAAAWN-TGCTA-AAAC-GRT-CTT---ACC--TGATAACA
11.Hydrophis platurus	MK775530	GTTATT-A-TA-GCCC-TTATT----AAGC-A-TAGCA-CTGAAAA--TGCTA-AATT-ACC-CC----AAT--TTATAACA
12.Hydrophis curtus	MK953549	GTTATC-A-TA-GCTT-ACAACC---AAGC-A-CAGCA-CTGAAAA--TGCTA-AGAC-AGC-CCA---ACT--TTATAACA
13.Emydocephalus ijimae	LC648431	GTTATT-A-TA-GCTT-ACAAAC---AAGC-A-TAGCA-CTGAAAA--TGCTA-AGAC-GGC-CCC---ACC--TAATAACA
14.Laticauda semifasciata	KY496325	GTTATT-A-TA-GCTT-ATAATC---AAGC-A-TAGCA-CTGAAAA--TGCTA-AGAT-GGT-CTAC--TCC--TGATAACA
15.Laticauda colubrina	KY496324	GTTGTC-A-TA-GCTT-AAAATC---AAGC-A-TAGCA-CTGAAAA--TGCTA-AGAT-GGC-CCAC--TCC--TGATAACA
16.Laticauda laticaudata	KY496323	GTTATT-A-TA-GCTT-ATATC----AAGC-A-TAGCA-CTGAAAA--TGCTA-AGAT-GGT-CCAC--CCC--TAGTAACA
17.Sinomicrurus japonicus	LC648432	GTTATC-A-TA-GCTT-ACTTTC---AAGC-A-TAGCA-CTGAAAA--TGCTA-AGAC-GGC-CTA---CCC--TGATAACA
18.Sinomicrurus peinani	MZ230594	GTTATC-A-TA-GCTT-ACCTTC---AAGC-A-TAGCA-CTGAAAA--TGCTA-AGAC-GGC-CCT---ACC--TGATAACA
19.Sinomicrurus mecclellandi	MT547176	GTTATC-A-TA-GCTT-ACAATC---AAGC-A-TAGCA-CTGAAAA--TGCTA-AGAC-GGC-CTC---CCC--TGATAACA
20.Micrurus fulvius	GU045453	GCTATC-A-TA-GCTT-ACCTTC---AAGC-A-TAGCA-CTGAAAA--TGCTA-AGAC-GGC-CT----TCC--TGATAGCA
21.Ophiophagus hannah	EU921899	GTTATC-A-TA-GCTT-ACACTC---AAGC-A-TAGCA-CTGAAAA--TGCTA-AGAC-GGC-CCC---CCC--TGATAACA
22.Naja atra	EU913475	GTTGTC-A-TA-GCTT-ACCTATC--AAGC-A-TAGCA-CTGAAAA--TGCTA-AGAC-GGC-CAT---GCC--TGACAGCA
23.Bungarus multicinctus	EU579522	GT-ACT-G-TA-GCTT-ACGATTA--AAGC-A-TAGCA-CTGAAAA--TGCTA-AGAC-GGC-ACTC--GCC--TGGTAACA
24.Bungarus fasciatus	EU579523	GTTATT-A-TA-GCTT-ACTATC---AAGC-A-TAGCA-CTGAAAA--TGCTA-AGAC-GGC-ATTC--ACC--TGATAACA
25.Sibon nebulatus	NC013985	GTTATT-A-TA-GCTT-ACCTTA---AAGC-G-TAGCA-CTGAAAA--TGCTA-AGAC-GGC-ACAGC-CTGA-TC-TAACA
26.Lycodon semicarrinatus	AB008539	GTTATC-A-TA-GCTT-ACGAATTA-AAGC-A-TAGCA-CTGAAAA--TGCTA-AGAT-GGT-CTAC--ACC--TGATTTAA
27.Ovophis okinavensis	AB175670	GTTGTCTG-TA-GCTT-AAGCCTA--AAGT-A-TAGCA-CTGAAAA--TGCTA-AGAT-GGT-CCA---ACC--CTCCAACA
28.Python regius	AB177878	GTTATT-G-TA-GCTT-ACACCA---AAGC-A-CAGCA-CTGAAAA--TGCTG-AGAC-GGT-ATTAC-ACCC-AAATAACA

Supplementary figure S5. Sequence comparison of tRNA^{Phe} genes among 21 snake species based on their clover-leaf secondary structures. *Hydrophis ornatus* mtDNA encodes three copies of tRNA^{Phe} genes (noted with 1-3) that correspond to F_1-F_3 in Fig. 3, respectively. *Hrdrophis melanocephalus* mtDNA encodes six copies of tRNA^{Phe} genes (noted with 1-6) that correspond to F_1-F_6 in Fig. 3, respectively. The red line indicates the anticodon.

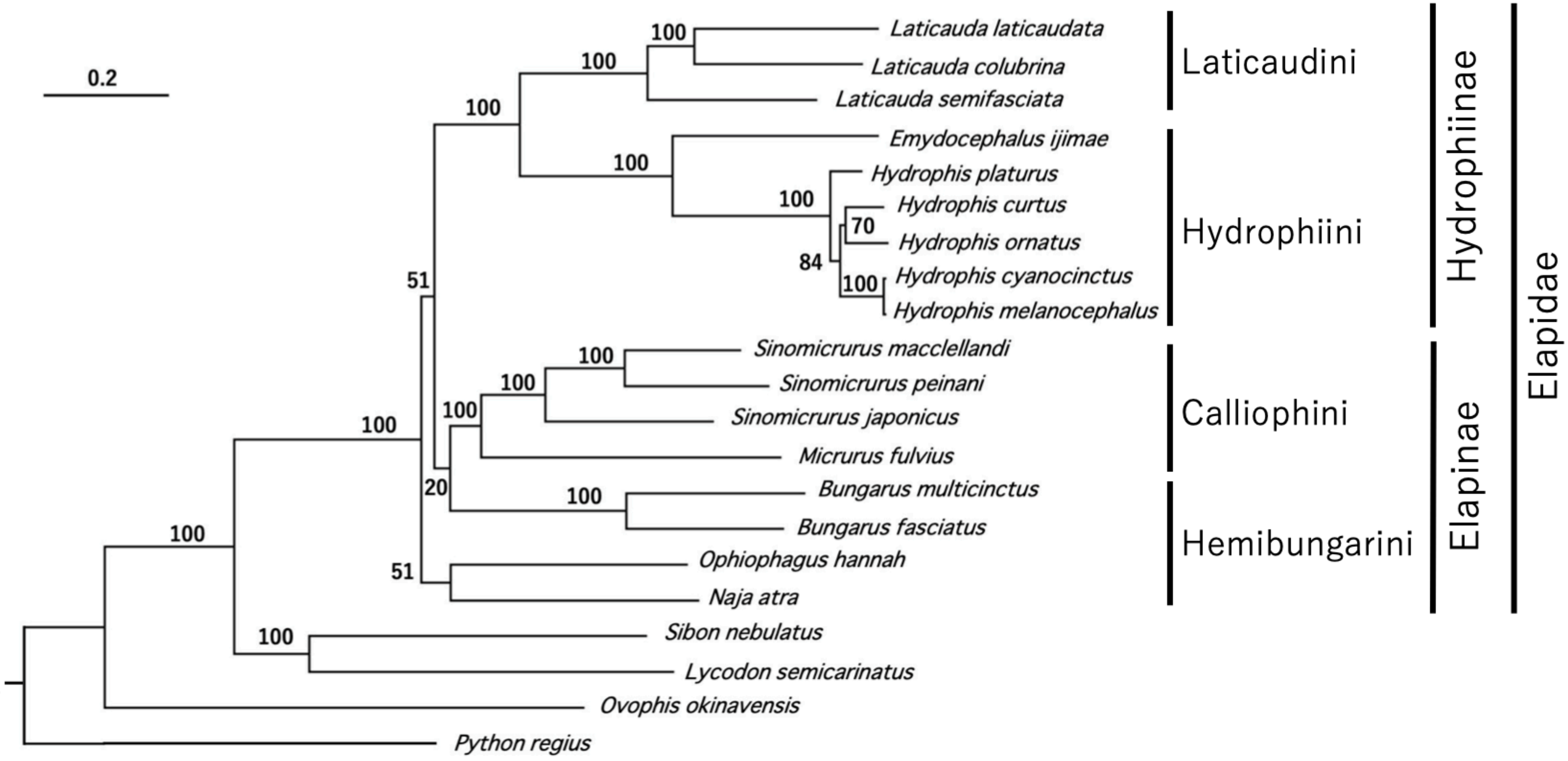

Supplementary figure S6. Maximum likelihood tree using nucleotide sequences of all 37 mitochondrial genes. Numbers at each node represent bootstrap probabilities from 10000 replications. Note that the monophyly of Hemibungarini is not seen in this tree and inter-species relationships within Hydrophiini in this tree are different from those in Fig. S7.

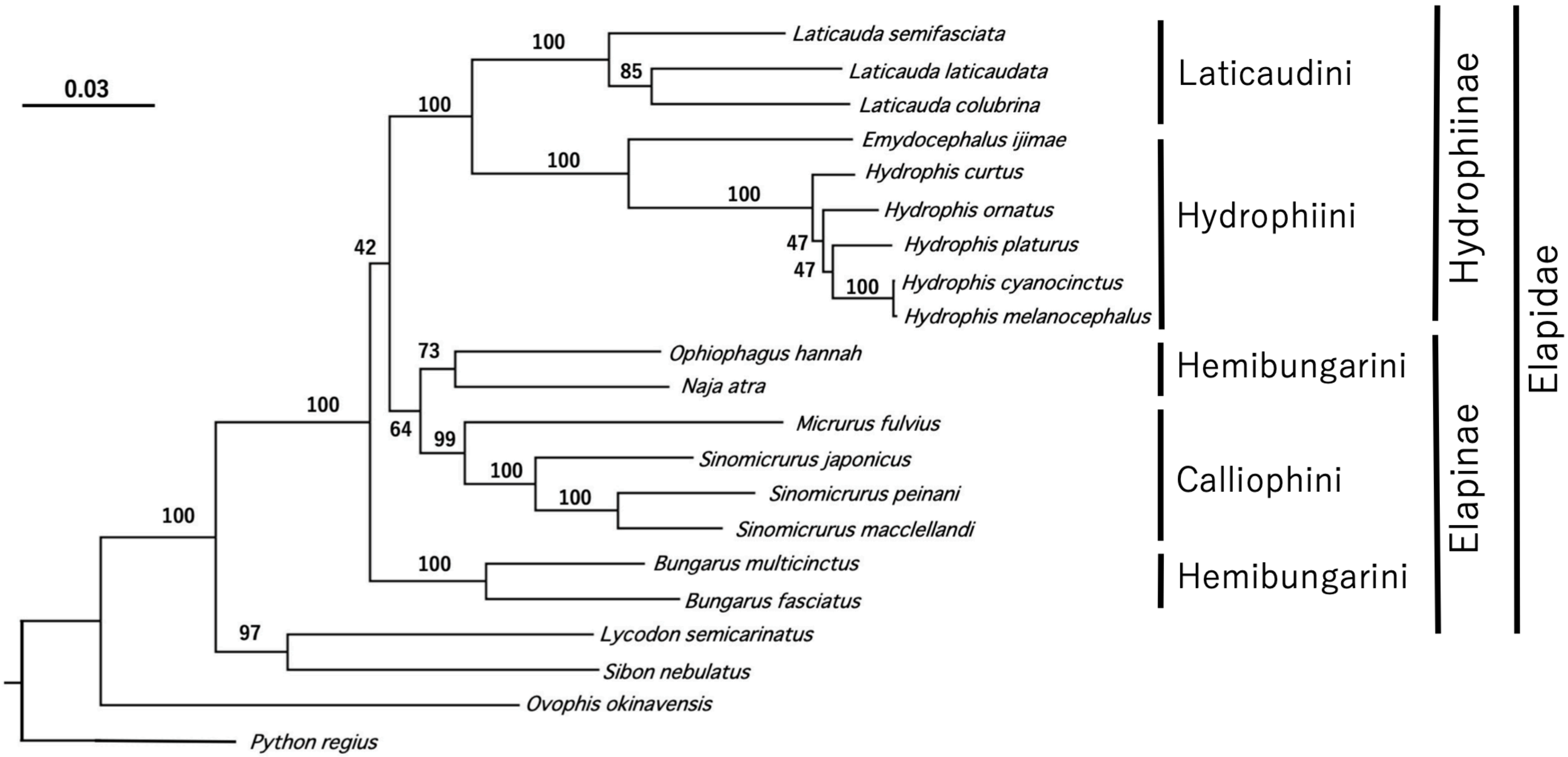

Supplementary figure S7. Maximum likelihood tree using amino acid sequences of 13 mitochondrial protein genes. Numbers at each node represent bootstrap probabilities from 5000 replications. Note that the monophyly of Hemibungarini is not seen in this tree and inter-species relationships within Hydrophiini in this tree are different from those in Fig. S6.
